# Supplementary material for: Behavioral shifts mask the success of legislation and outreach for endangered species recovery
Source: Nat Commun. 2026 Mar 18;17:1819. doi: 10.1038/s41467-026-69617-4 (PMC13000190; doi:10.1038/s41467-026-69617-4)
Supplement: Supplementary file 1 — Supplementary Information [file 41467_2026_69617_MOESM1_ESM.pdf]

Supplementary Information for:

Behavioral shifts mask the success of legislation and outreach for endangered species recovery

Victoria J. Bakker[1*#], Daniel F. Doak[2], Alacia Welch[3], L. Joseph Burnett[4], María C. Porras Peña[5], Joseph Brandt[6], Sharon A. Poessel[7], Steve Kirkland[6], Rachel Wolstenholme[3], Daniel Ryan[3], Mike Stake[4], Arianna Punzalan[6], Nacho Vilchis[8], Melissa A. Braham[9], Myra E. Finkelstein[10*#]

Affiliations

[1] Department of Ecology, Montana State University, Bozeman, MT, USA

[2] Department of Environmental Studies, University of Colorado, Boulder, CO, USA

[3] National Park Service, Pinnacles National Park, Paicines, CA, USA

[4] Ventana Wildlife Society, Monterey, CA, USA

[5] Parque Nacional Sierra de San Pedro Mártir, Ensenada, Baja California, Mexico

[6] US Fish and Wildlife Service, Ventura, CA, USA

[7] U.S. Geological Survey, Forest and Rangeland Ecosystem Science Center, Boise, ID, USA

[8] Conservation Science & Wildlife Health, San Diego Zoo Wildlife Alliance, San Diego, CA, USA

[9] Conservation Science Global, Inc., Cape May, NJ, USA

[10] Microbiology and Environmental Toxicology Department, University of California, Santa Cruz, CA, USA

[*] Corresponding authors: vjbakker@gmail.com, myraf@ucsc.edu

[#] Equally contributing first authorship

Contents

Notes

Supplementary Note 1 – Effects of outreach on blood lead exposure and links to survival

Supplementary Note 2 – Predictors of survival

Supplementary Note 3 – Parameterizing the population growth model

Figures

Supplementary Fig. 1 – Ranging and foraging behavior for California condor (*Gymnogyps californianus*) flocks included in study

Supplementary Fig. 2 – Tag returns for deer and wild pig hunting and nonlead outreach activities in California

Supplementary Fig. 3 – Variable importance metrics for linear mixed models (LMMs)

Supplementary Fig. 4 – Apparent trends in wild pig culling in California condor (*Gymnogyps californianus*) foraging areas

Supplementary Fig. 5 – Outreach decreased lead exposure for California condors (*Gymnogyps californianus*) in California

Supplementary Fig. 6 – The number of people contacted (*Contacts*) and boxes of ammunition distributed (*Boxes*) increased survival for *Central* California condors (*Gymnogyps californianus*) across a range of conditions

Supplementary Fig. 7 – The number of people contacted (*Contacts*) and boxes of ammunition distributed (*Boxes*) increased survival for *Southern* California condors (*Gymnogyps californianus*) across a range of conditions

Tables

Supplementary Table 1 – Variables used in analyses of California condors (*Gymnogyps californianus*)

Supplementary Table 2 – Age- and stage-based matrix model for California condors (*Gymnogyps californianus*) – model structure

Supplementary Table 3 – Predictors of blood lead levels for California condors (*Gymnogyps californianus*) in California

Supplementary Table 4 – Predictors of contaminated meal exposure rates for California condors (*Gymnogyps californianus*) in California

Supplementary Table 5 – Predictors of blood lead levels for California condors (*Gymnogyps californianus*) in California when outreach was occurring

Supplementary Table 6 – Predictors of survival for California condors (*Gymnogyps californianus*) in California.

Supplementary Table 7 – Age- and stage-based matrix model for *Central* and *Southern* – parameter values

Supplementary Table 8 – Age- and stage-based matrix for *Baja* – parameter values

Supplementary Table 9 – Model selection table for predictors of lead exposure for California condors (*Gymnogyps californianus*) in *Central*

Supplementary Table 10 – Model selection table for predictors of lead exposure for California condors (*Gymnogyps californianus*) in *Southern*

Supplementary Table 11 – Model selection table for predictors of lead exposure for California condors (*Gymnogyps californianus*) in *Central* including outreach metrics

Supplementary Table 12 – Model selection table for predictors of lead exposure for California condors (*Gymnogyps californianus*) in *Southern* including outreach metrics

Supplementary Table 13 – Model selection table for predictors of overall survival ($S_{Overall}$) for California condors (*Gymnogyps californianus*)

Supplementary Table 14 – Model selection table for predictors of survival of lead mortality (S_{Pb}) for California condors (*Gymnogyps californianus*)

Supplementary Table 15 – Model for reproduction of California condors (*Gymnogyps californianus*) in California and Baja California, Mexico used in population model.

Supplementary Table 16 – Survival model for California condors (*Gymnogyps californianus*) in California and Baja California, Mexico used in population model.

<u>Data</u>

Supplementary Data 1 – Input data and code. Includes:

1. InputDataCACO.xlsx. Excel file containing input data needed to run all analyses. Data are included in separate tabs:

- VarDefs (variable definitions for all data in xlsx file),
- PbDataCaMx (blood lead levels for Central, Southern and Baja),
- PbFeatherDataCaMx (feather lead levels for Central, Southern and Baja),
- PbDataCa (blood lead levels for Central and Southern, with covariates),
- sAllCaMx (input data for overall survival, sOverall, analyses for Central, Southern and Baja),
- PbCaMx (input data for lead survival, sPb, analyses for Central, Southern and Baja),
- sOtherKnownCaMx (input data for survival of other known causes of death to correct sPb analyses for Central, Southern and Baja),
- sMIWUndCaMx (input data for survival of unknown causes of death to correct sPb analyses for Central, Southern and Baja),
- sAllCa (input data for overall survival, sOverall, analyses for Central and Southern, with covariates),
- sPbCa (input data for lead survival, sPb, analyses for Central and Southern, with covariates),
- reproAll (input data for reproductive rate analyses for Central, Southern, and Baja),
- DeerHuntBiMo (bimonthly deer tag reports),
- PigHuntBiMo (bimonthly pig tag reports,
- PigCullMo (monthly pig cull reports),
- Nonlead (nonlead outreach effort data)

2. PbAndSurvBasicTrends.Rmd. R notebook that calculates basic trends in blood lead exposure by flock and time period and feather lead by flock and basic trends in overall and lead survival by flock and year.

3. PbExposureLmmAnalyses.Rmd. R notebook that runs all linear mixed models analyzing drivers of lead exposure and also extrapolates effects of outreach on lead exposure to survival.

4. SurvReproPvaAnalyses.Rmd. R notebook that runs analyses of drivers of overall and lead survival. This notebook also estimates survival and reproduction to parameterize age- and stage-based matrix models to generate population growth rates for the Southern, Central, and Baja flocks.

5. SetupPGJagsModels.Rmd. R notebook that sets up Poisson Gamma models to analyze meal contamination rates using the R script DoubleEquationplus.jags.condors.R.

6. DoubleEquationplus.jags.condors.R. R script that contains the jags code to run analyses of meal contamination rates for Southern and Central flocks using Poisson-Gamma model.

Supplementary Note 1 – Effects of outreach on blood lead exposure and links to survival

For outreach analyses, we compiled metrics for outreach statewide (*BoxesStatewide* and *ContactsStatewide*), and also for flock-specific outreach in the high-use foraging counties for each flock (Supplementary Fig. 1b, c, *BoxesFlock* and *ContactsFlock*). For these analyses, we restricted our data to the subset of years outreach occurred and data were compiled (*Bioyears* 2012 – 2021 for *Central*, 2014 – 2021 for *Southern*). For both flocks, *BoxesFlock* and *ContactsStatewide* were most strongly associated with condor *BloodLead*. Thus, for final model-building, we defined *Boxes* as flock-specific variables and *Contacts* as statewide variables (Supplementary Table 1). *Post2008Ban* was not considered in outreach analyses because the analysis included no data prior to 2008. To reduce the number of variables due to smaller sample sizes, we excluded the behavioral variable *Presence* from outreach analyses as it showed a weaker relationship with *BloodLead* than did *Proffered*. We excluded *Targeted* for *Southern* analyses because the outreach dataset had no *Targeted* samples in this time frame. Finally, *PigHunt* in *Southern* in 2014 was 50% higher than all other years for the outreach dataset and models fit with this year produced implausibly high lead levels at high *PigHunt*. Consequently, we used a modified variable, *PigHuntCeil*, which plateaued such that all values >1.0 were assigned a value of 1.0 (Supplementary Table 1).

The best *Central* outreach model (Supplementary Tables 5a, 11, Supplementary Fig. 5a-d) to predict *BloodLead* included the same major effects supported in models using the full dataset and added three interacting effects: negative interactions of *DeerHunt* with both *Boxes* and *Contacts* and a negative interaction of *PigHunt* and *Contacts*, indicating high outreach reduced the effect of high hunt levels on condor blood lead levels.

Similarly, the best *Southern* outreach model (Supplementary Tables 5b, 12, Supplementary Fig. 5e-h) to predict *BloodLead* generally included the same major effects as supported when modeling the full dataset, although *PigHuntCeil:Post2019Ban* replaced *PigHunt:Post2008Ban*. The *Southern* outreach model added *Contacts* and *Boxes* and a negative interaction between *Contacts* and both *DeerHunt* and *PigHunt*, indicating high *Contacts* reduced the effect of high hunt levels on condor blood lead levels. The *Southern* model also included a positive interaction between *Boxes* and *PigHunt*, indicating the effect of *Boxes* declined as *PigHunt* increased.

Combining our outreach models to predict *BloodLead* and our Cox models to predict 56-day survival following *BloodLead* measurements, we predicted that outreach increased 56-day survival in the peak of the deer season in *FallWinter* by at least 0.5% and up to almost 10% for both flocks (Supplementary Figs. 6-7). For *Central*, both *Boxes* and *Contacts* were predicted to increase survival for all observed levels of outreach and hunting. For *Southern*, the level of pig hunting had more influence; in seasons with high *PigHunt*, *Contacts* were more important while in seasons with low *PigHunt*, *Boxes* were more important. This reflects the reduction in effectiveness of *Boxes* at high pig hunt levels in *Southern*, perhaps due to a saturation effect. Cox survival models did not violate the proportional hazards assumption for either flock ($P \geq 0.16$).

Although we assume—and our models indicate—that higher outreach is associated with higher use of nonlead ammunition, future studies are needed to validate this assumption. In

addition, since our outreach data started after the first ban (2008), we only analyzed the effects of outreach occurring after the implementation of lead bans. Therefore, our results assume the presence of a legislative ban and do not necessarily apply to the effectiveness of outreach when nonlead ammunition use is voluntary.

Supplementary Note 2 – Predictors of survival

Although we have previously documented some sex differences in survival[1], we did not consider sex as a predictor of survival in these analyses because our goal was to identify behavioral and threat-related factors that drive mortality, especially those related to lead. Including sex in survival models does not qualitatively change the strength or significance of other model effects.

For S_{Pb} (Supplementary Table 14), we selected the second-ranked model based on AICc, which substitutes CECA:PigHunt:Post2019Ban for CECA:Post2019Ban, because this model was nearly as well supported as the top-ranked model (delta AICc 0.39) and provided more information on the processes driving survival in the time interval after the 2019 ban.

The best model (Supplementary Table 6a) to predict $S_{Overall}$ (Supplementary Table 13) and the best model (Supplementary Table 6b) to predict S_{Pb} (Supplementary Table 14) showed patterns that reinforced *BloodLead* LMMs. Consistent with its strong negative association with lead exposure, *Proffered* was strongly positively associated with $S_{Overall}$ and S_{Pb}, and *Presence* was also positively associated with $S_{Overall}$ in the *Southern* flock. These effects indicate that condors had higher survival if they had a high rate of proffered feeding or were frequently detected by biologists within the management area. In addition, *Coastal* was positively associated with S_{Pb} in *Central*, the only flock where marine mammal feeding has been observed[2]. The relationship between *Proffered* and lead mortality did not differ between the flocks despite the lower overall rate in *Southern* (Supplementary Fig. 1d, e).

The effects of hunting on $S_{Overall}$ and S_{Pb} were similar to hunting effects on condor lead exposure (*BloodLead*), with wild pig hunting decreasing condor survival in *Central* and exerting increasing negative effects through time. Likewise, deer hunting increased both $S_{Overall}$ and S_{Pb} in *Southern* after the 2019 ban ($S_{Overall}$) or after the 2008 ban (S_{Pb}).

As with previous analyses[1,3], we found that $S_{Overall}$ was lower for newly fledged and newly released birds, which we attribute to lack of familiarity with the environment. At the same time, S_{Pb} was higher for condors in their first two years and both S_{Pb} and $S_{Overall}$ were lower for the young adult age class. Vulnerability at the young adult age class is not the result of behavior changes with age (Supplementary Fig. 1d-h) but may arise due to a selective loss of birds that are physiologically more vulnerable to lead toxicity combined with the compounding effects of cumulative lead exposures[4].

Supplementary Note 3 – Parameterizing the population growth model

To parameterize survival rates in the population matrix model, we built a model to predict $S_{Overall}$ without behavioral variables. In the best model (Supplementary Table 16), survival differed by flock, with *Baja* having higher survival than *Central* and *Southern*, which had the same survival rate. First year birds and birds released less than one year had lower survival, as did females. We also included factors to account for different survival when release sites were new (< 6 years since first releases).

To parameterize reproductive rates in the population matrix model, we built a model to predict the probability of a female successfully fledging a chick (*Succ*). The best model (Supplementary Table 15) indicated there was no difference in *Succ* by flock. *Succ* increased with age up until age 9 (*Ageto9*), with a separate *Age5* effect to account for the extremely low probability of breeding at this age. *Succ* was lower during the *EarlyBreedingYrs*, or when breeding age birds were present in a flock for 7 years or fewer. Females that spent a greater proportion of the previous 180 days free-flying were more likely to fledge a chick. Breeders who successfully bred the previous year had the lowest probability of fledging a chick while breeders who failed the previous year had the highest probability.

Based on these reproduction and survival models, we used the following parameter estimates for our matrix model for *Central* and *Southern* (Supplementary Table 7) and *Baja* (Supplementary Table 8):

Reproduction	***Survival***
<u>*All flocks*</u>	<u>*Central* and *Southern*</u>
$fem = 0.5$	$S_{FirstYear} = 0.835$
$Succ_{NB5} = 0.010$	$S_{AfterFirstYear} = 0.909$
$Succ_{NB6} = 0.096$	
$Succ_{NB7} = 0.126$	<u>*Baja*</u>
$Succ_{NB8} = 0.165$	$S_{FirstYear} = 0.907$
$Succ_{NB9on} = 0.212$	$S_{AfterFirstYear} = 0.951$
$Succ_{FB7} = 0.196$	
$Succ_{FB8} = 0.250$	
$Succ_{FB9on} = 0.312$	
$Succ_{SB6} = 0.025$	
$Succ_{SB7} = 0.035$	
$Succ_{SB8} = 0.046$	
$Succ_{SB9on} = 0.062$	

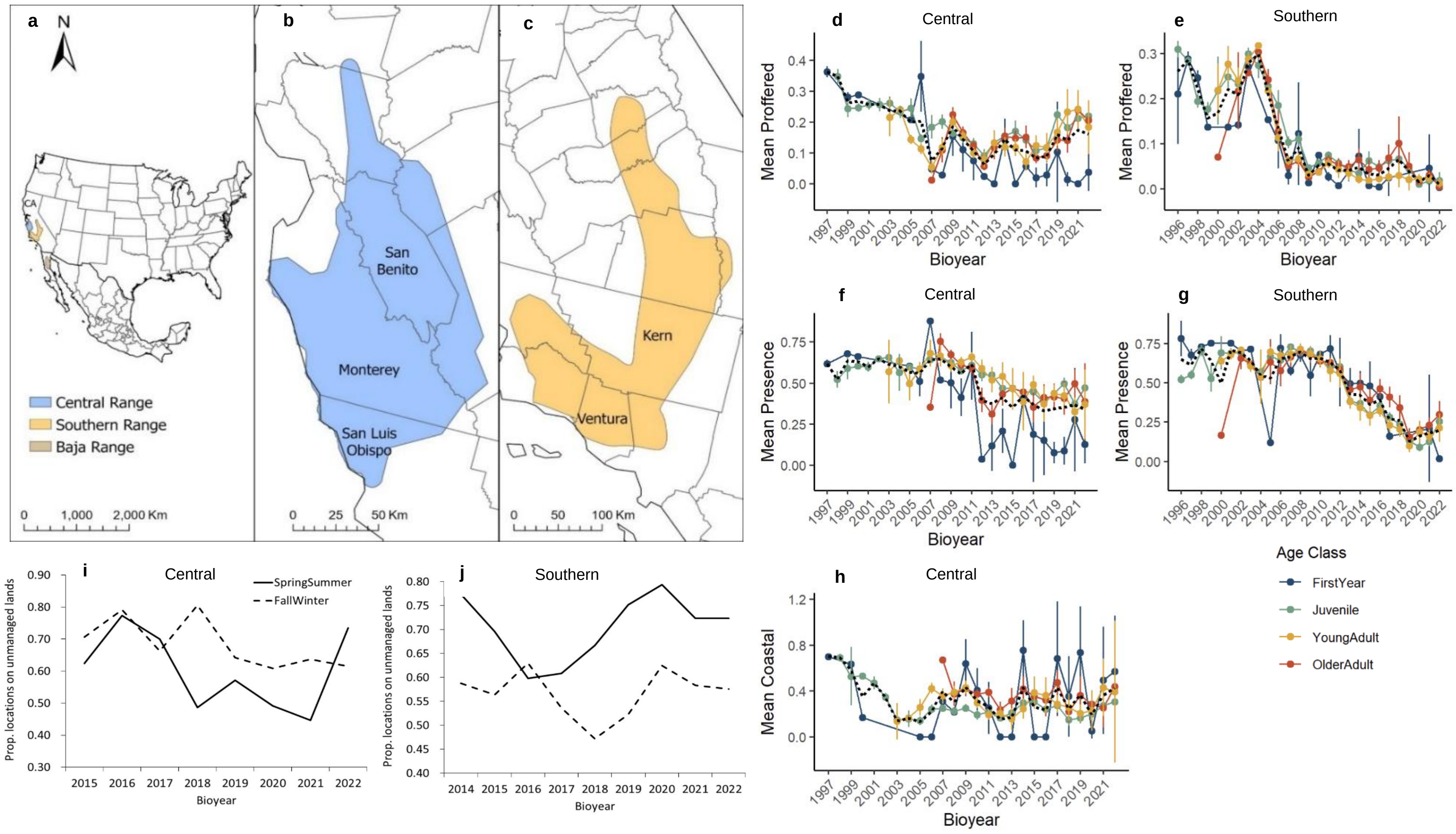

Supplementary Fig. 1. Ranging and foraging behavior for California condor (*Gymnogyps californianus*) flocks included in study. a Condor flocks were *Central*, jointly managed by Pinnacles National Park and Ventana Wildlife Society, *Southern*, managed by U.S. Fish and Wildlife Service, and *Baja*, managed by Comisión Nacional de Áreas Naturales Protegidas and Espacios Naturales y Desarrollo Sustentable. Polygons shown in **a – c** were generated based on all 2023 – 2024 GPS locations, excluding outliers. Condor ground locations, assumed to be foraging locations, were highly concentrated in a few counties, which were relatively constant through time. We identified high-use foraging counties for **b** *Central* as Monterey, San Benito, and San Luis Obispo and for **c** *Southern* as Kern and Ventura based on the mean proportion of ground locations located in each county. Proportional use averaged across *Bioyears* and seasons for *FallWinter* (Sep – Feb) and *SpringSummer* (Mar – Aug) was, for *Central*, 0.26 and 0.33 in Monterey, 0.37 and 0.45 in San Benito, and 0.35 and 0.18 in San Luis Obispo, and, for *Southern*, 0.77 and 0.76 in Kern and 0.20 and 0.16 in Ventura. **d – h** As flock ranges expanded in California, condor behaviors have shifted (age-specific mean behaviors and 90% confidence intervals shown as solid lines, overall mean behaviors shown as dashed lines). Proffered feeding (*Proffered)* and presence in the management area (*Presence)* decreased for both flocks and time in the coastal area (*Coastal)* decreased for *Central*. Sample sizes

were *n* = 52, 178, 79, 40 in *Central* and *n* = 45, 161, 78, 51 in *Southern* for *FirstYear*, *Juvenile*, *YoungAdult*, and *OlderAdult* condors. **i – j** Condors also exhibited seasonal variation in space use as evidenced by variation in the proportion of ground locations on unmanaged lands, calculated using ArcGIS Pro to associate locations with the California Protected Areas Database (https://data.cnra.ca.gov/dataset/california-protected-areas-database) by flock, season, and *Bioyear*, then summing locations outside of protected areas and dividing by the total number of ground locations. We restricted data to season-*Bioyear* combinations with ≥10 condors tracked (*Central*, *FW: n* = 13 – 19, *SS: n* = 11 – 15; *Southern*, *FW: n* = 25 – 82, *SS: n* = 30 – 76). See Supplementary Table 1 for variable and age class definitions.

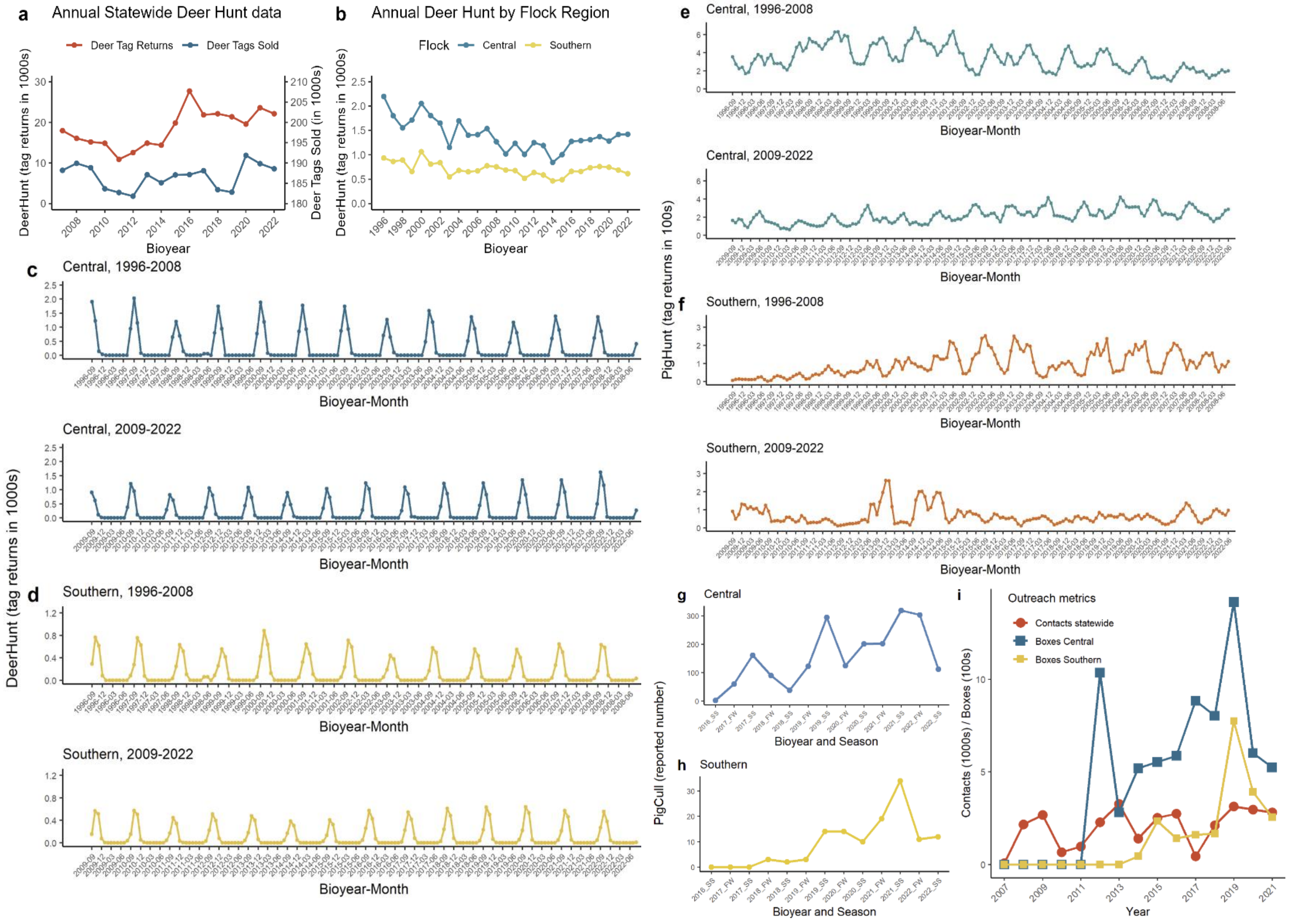

Supplementary Fig. 2. Tag returns for deer and wild pig hunting and nonlead outreach activities in California. a Annual (i.e., *Bioyear*, Sep – Aug) statewide deer tag returns before and after the 2014 changes in reporting requirements (note different scales for the two vertical axes). The remaining plots show California condor (*Gymnogyps californianus*) flock-specific data, summarized for high-use foraging counties (see Supplementary Fig. 1b, c and Methods: Condor space use): **b** Annual deer tag returns for *Central* and *Southern*, with 1996 – 2014 adjusted based on a correction factor of 1.53, **c** *Central* and **d** *Southern* bimonthly (i.e., sum of current and previous month) deer tag returns (*DeerHunt),* **e** *Central* and **f** *Southern* bimonthly wild pig tag returns (*PigHunt)*, **g** *Central* and **h** *Southern* wild pig cull reports by season (*FallWinter*, *FW*: Sep – Feb, *SpringSummer*, *SS*: Mar – Aug) from *Bioyears* 2016 – 2022, and **i** outreach metrics, or the number of people contacted statewide (*Contacts*) and the number of boxes of ammunition distributed in condor high-use foraging counties (*Boxes Central* and *Boxes Southern*, note different scales for *Contacts* and *Boxes* on the vertical axis). We used calendar year outreach effort to predict condor blood lead levels for the *Bioyear*. For example, we assumed boxes distributed Jan 2012 – Dec 2012 would predict blood lead Sep 2012 – Aug 2013 (*Bioyear* 2012). *Contacts* exclude contacts directly associated with receiving a box of ammunition. We restricted analyses to years when both *Contacts* and *Boxes* occurred, or *Bioyears* 2012 – 2021 for *Central*, and 2014 – 2021 for *Southern*. See Supplementary Table 1 for variable definitions.

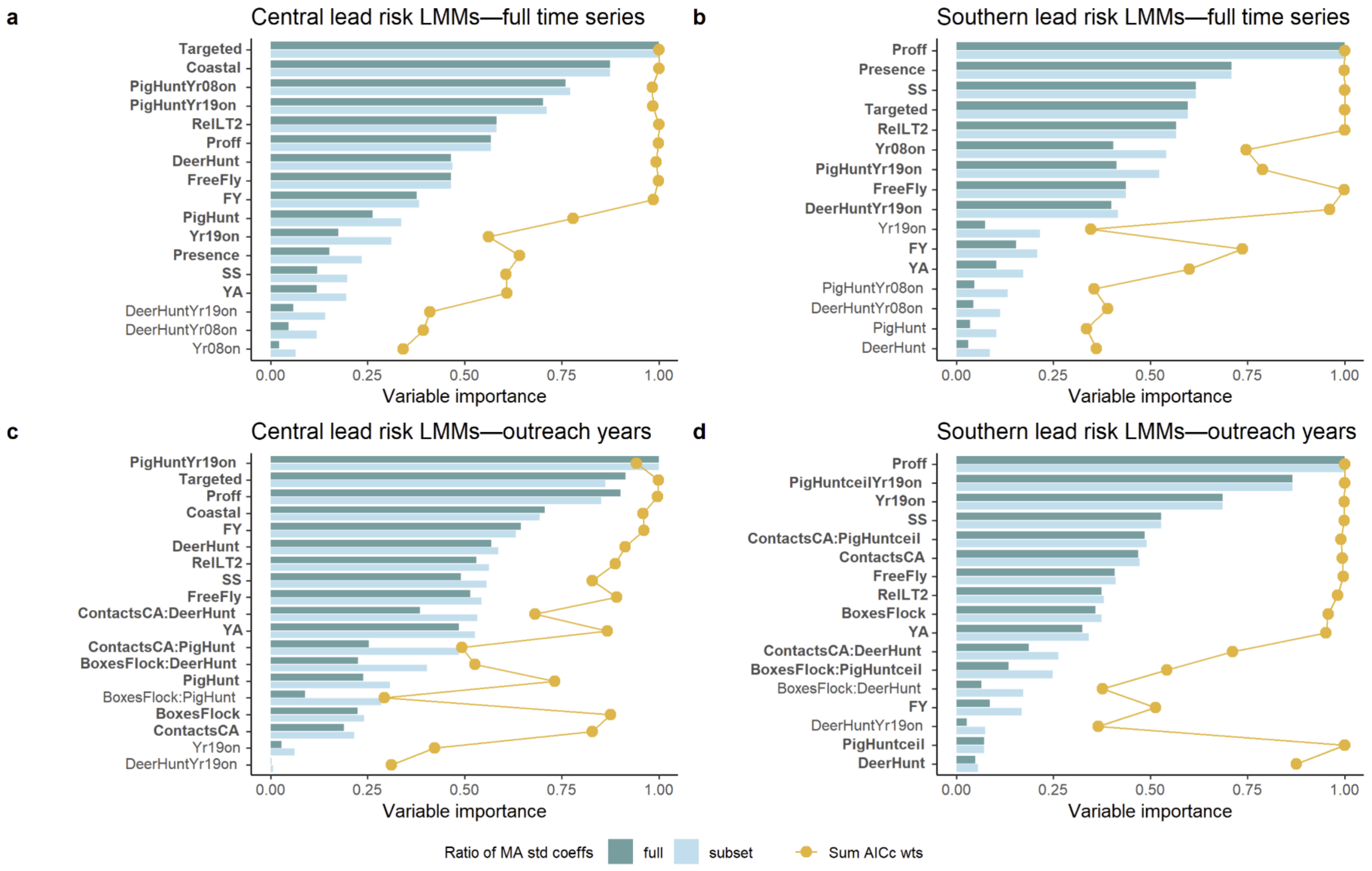

Supplementary Fig. 3. Variable importance metrics for linear mixed models (LMMS). Because there are limitations associated with most approaches to quantifying variable importance, especially in the presence of interactions[5,6], we calculated three metrics (see Methods: Models to predict lead exposure). Variables included in the best-supported model for each analysis type are bolded on the y-axis (Supplementary Tables 9-12). Gold points are the sum of model AICc weights for all models containing the predictor variables. Horizontal bars use model-averaged coefficients for standardized variables (centered and multiplied by the partial standard deviation, which adjusts for multicollinearity among variables) and show the ratio of the absolute value of the coefficient to the maximum coefficient [5,6]. Dark blue bars model average using AICc weights for all models ("full"), while light blue bars use only the subset of models containing the variable ("subset"). We ordered variables by full ratio model-averaged standardized coefficients. The relative importance of variables is generally consistent across metrics, with most differences due to variables included in interactions, which is a recognized limitation of variable importance metrics (e.g., weak main effects will be included in models with highly supported interactions and thus will be highly weighted, main effects will scale differently in models with and without interactions). Numbers of models containing each variable were **a** *Central* lead risk models for the full time series (*Bioyears* 1996 – 2022): 65,536 of 131,072 models, **b** *Southern* lead risk models for the full time series (*Bioyears* 1996 – 2022): 32,768 of 65,536 models, **c** *Central* lead risk models for the outreach years (data available for *Bioyears* 2012 – 2021): 48,128 for all, except 69,632 for *DeerHunt*, *PigHunt*, *Boxes*, *Contacts* and 26,624 for interactions between these hunt and outreach variables, of 96,256 models, and **d** *Southern* lead risk models for the outreach years (data available for *Bioyears* 2014 – 2021): 12,034 for all, except 17,408 for *DeerHunt*, *PigHunt*, *Boxes*, *Contacts* and 6,656 for interactions between these hunt and outreach variables, of 24,064 models.

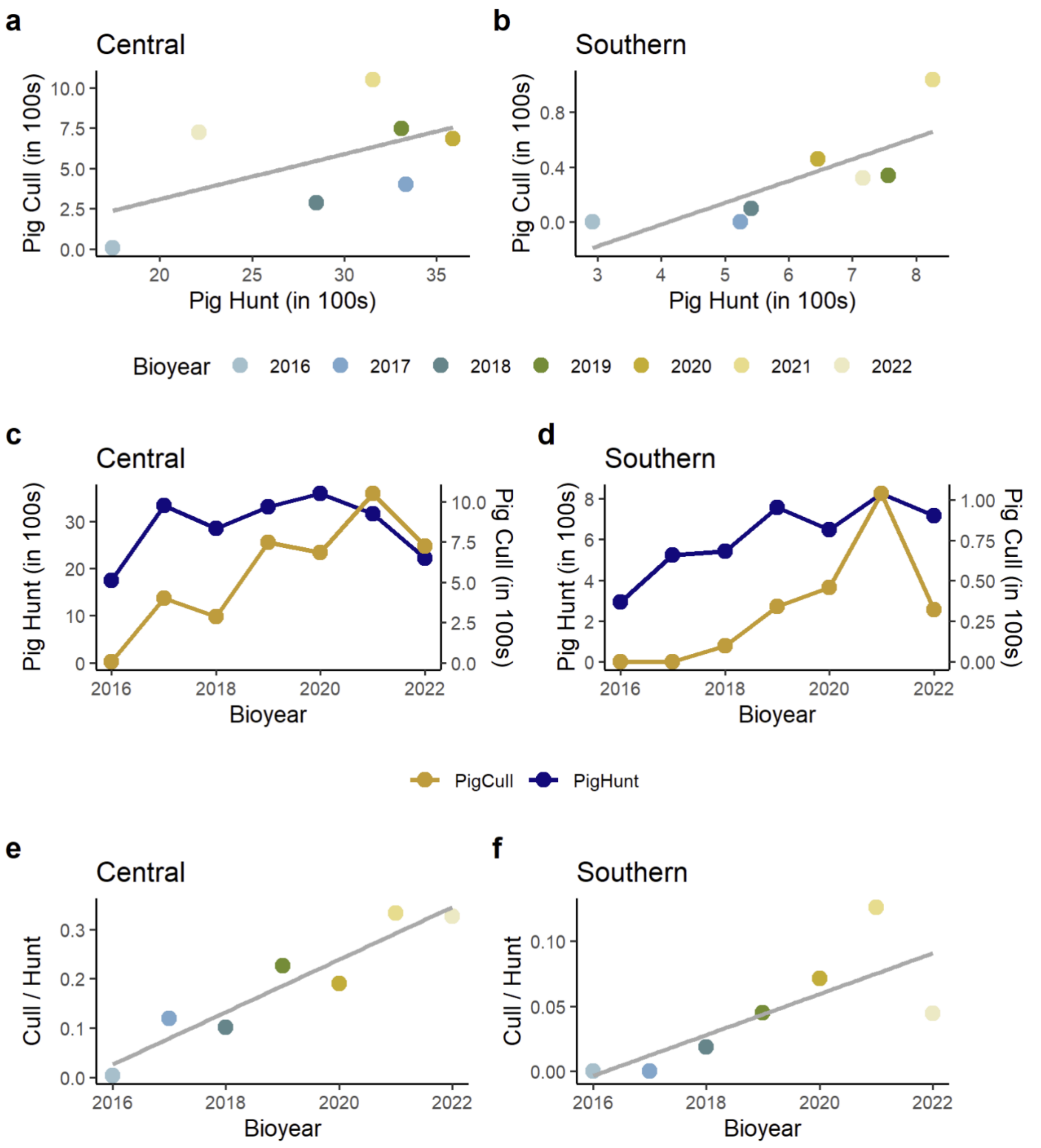

Supplementary Fig. 4. Apparent trends in wild pig culling in California condor (*Gymnogyps californianus*) foraging areas. Reliable data on numbers of wild pigs culled in California prior to *Bioyear* 2016 are lacking. Thus, we were unable to assess direct long-term relationships between pig culling and condor lead exposure. However, we hypothesize that pig hunt and pig cull levels are positively correlated and that pig culling is increasing relative to pig hunting likely due to an increasing wild pig population, especially in *Central* foraging areas. Available data for *Bioyears* 2016 – 2022 for the counties heavily used for foraging by condors (see Supplementary Fig. 1b, c and Methods: Condor space use) support this hypothesis. Pig cull levels (*PigCull*) were positively correlated with pig hunt levels (*PigHunt*) in **a, c** *Central* ($r = 0.54$, $P = 0.21$) and **b, d** *Southern* ($r = 0.79$, $P = 0.036$). In addition, there was strong support for a positive linear trend through time in the ratio of *PigCull* to *PigHunt* over this time period in both **e** *Central*: *PigCull/PigHunt* ~ -0.03 + 0.05*Trend*, adjusted $R^2 = 0.88$, $P = 0.001$ and **f** *Southern*: *PigCull/PigHunt* ~ -0.02 + 0.02*Trend*, adjusted $R^2 = 0.49$, $P = 0.049$. For all plots and analyses, *Bioyear* 2016 only includes data from Jan 2017 – Aug 2017 (vs Sep 2016 – Aug 2017) due to lack of culling data for 2016 and earlier.

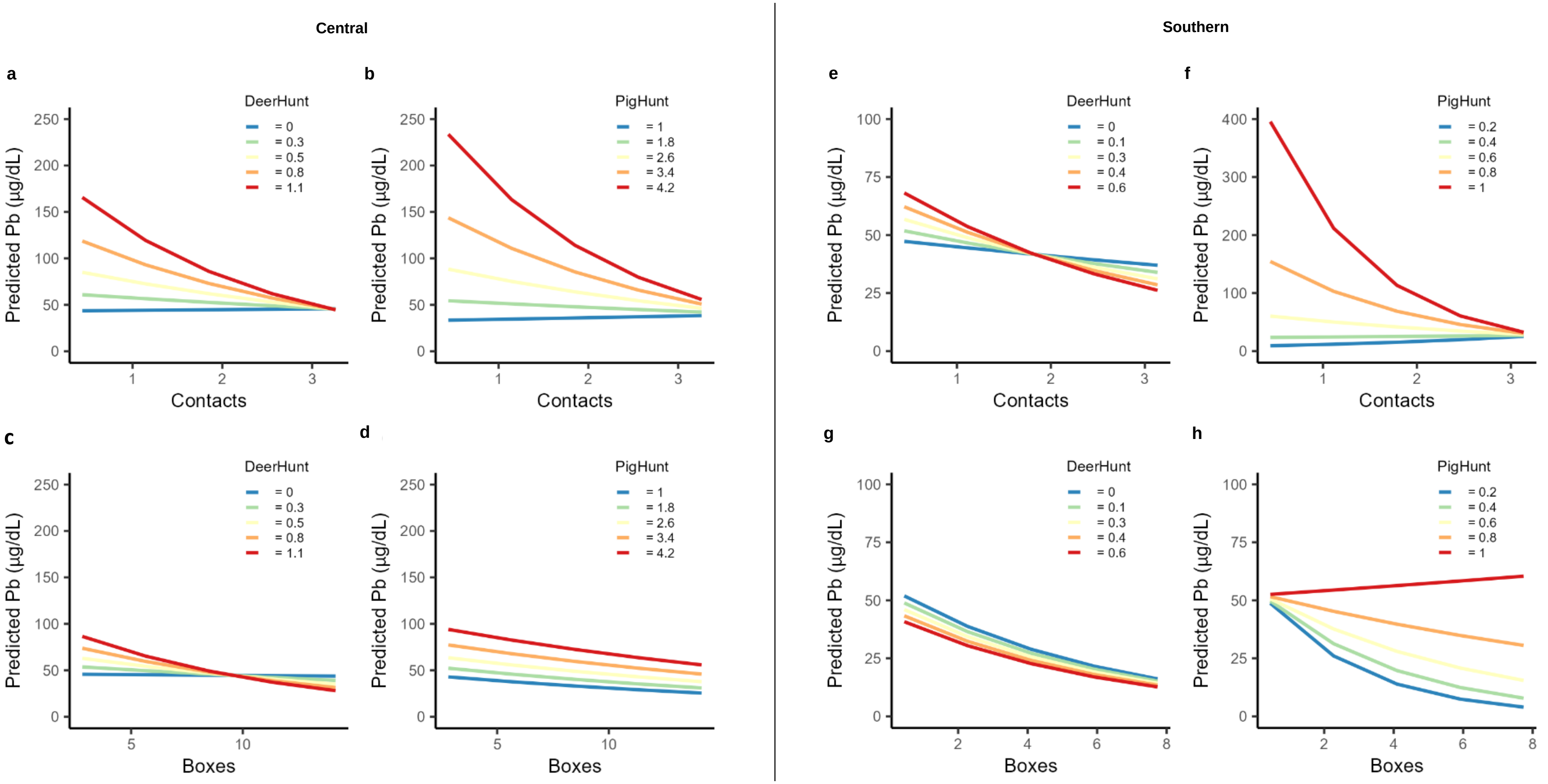

Supplementary Fig. 5. Outreach decreased lead exposure for California condors (*Gymnogyps californianus*) in California. Predicted blood lead levels as a function of outreach for **a – d,** *Central,* and **e – h,** *Southern. Contacts* for different deer hunt levels (**a, e**) and wild pig hunt levels (**b, f**), and as a function of *Boxes* for different deer hunt (**c, g**) and wild pig hunt levels (**d, h**). Plots are based on the best-supported models using data on *Contacts* and *Boxes* from *Bioyears* 2012 – 2021 for *Central* and *Bioyears* 2014 – 2021 for *Southern*, the time frames when both activities occurred (see Supplementary Fig. 2i). Most values not varied in plots are set at their medians. For plots that varied *PigHunt* (**b, d, f, h**), *DeerHunt* was set to the seasonal median for *FallWinter*. See Supplementary Note 1 for additional details, Supplementary Table 1 for variable definitions.

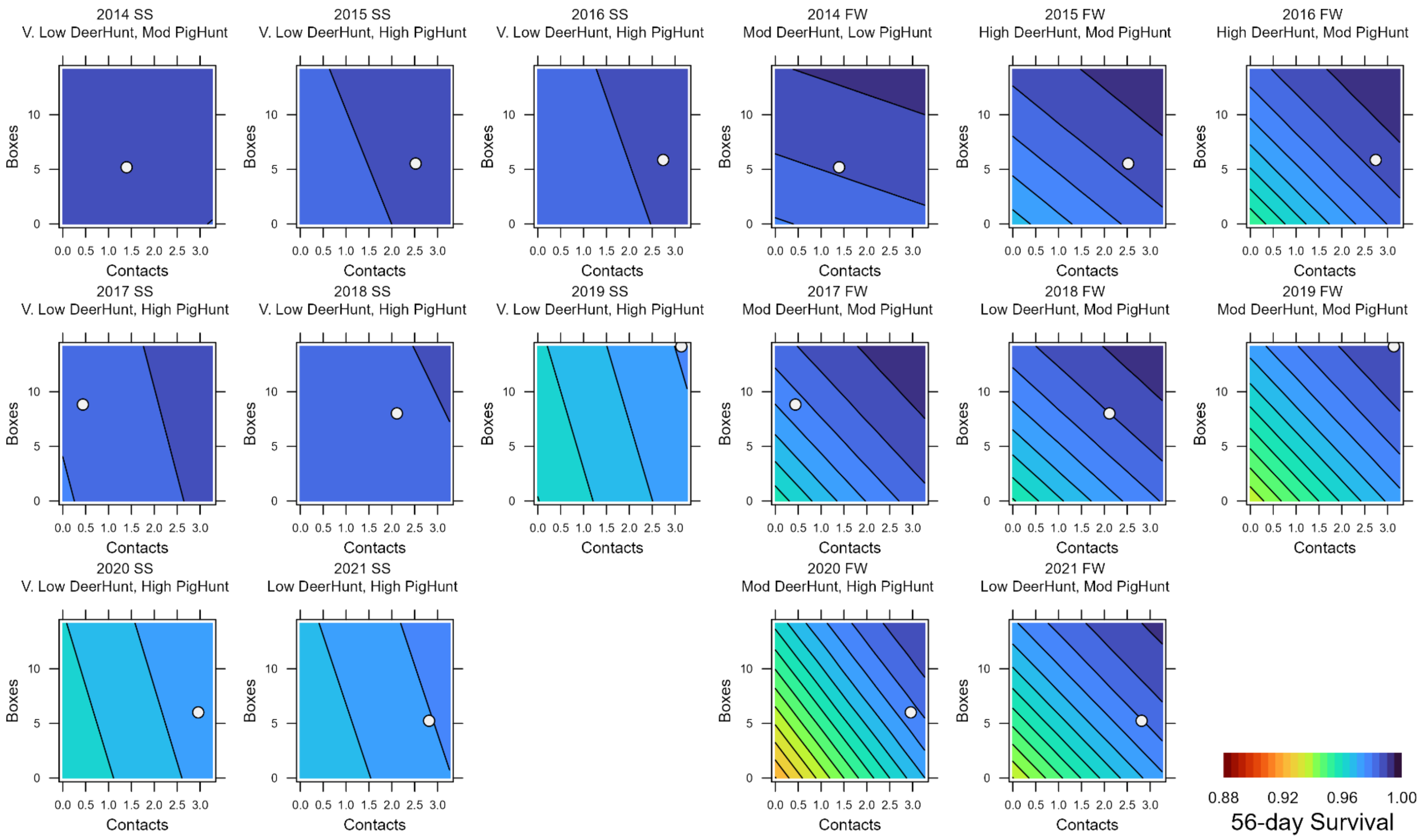

Supplementary Fig. 6. The number of people contacted (*Contacts*) and boxes of ammunition distributed (*Boxes*) increased survival for *Central* California condors (*Gymnogyps californianus*) across a range of conditions. Plots show predicted 56-day survival for an older adult condor for different combinations of statewide *Contacts* and regional *Boxes* with most model predictors set at their season-specific means (*FallWinter*, *FW* and *SpringSummer*, *SS*). For *FallWinter*, we set *DeerHunt* at its mean for the peak deer hunting months of Sep and Oct. Observed outreach effort (*Contacts* and *Boxes*) for the specific year are plotted (white dot). See Supplementary Note 1 for additional details.

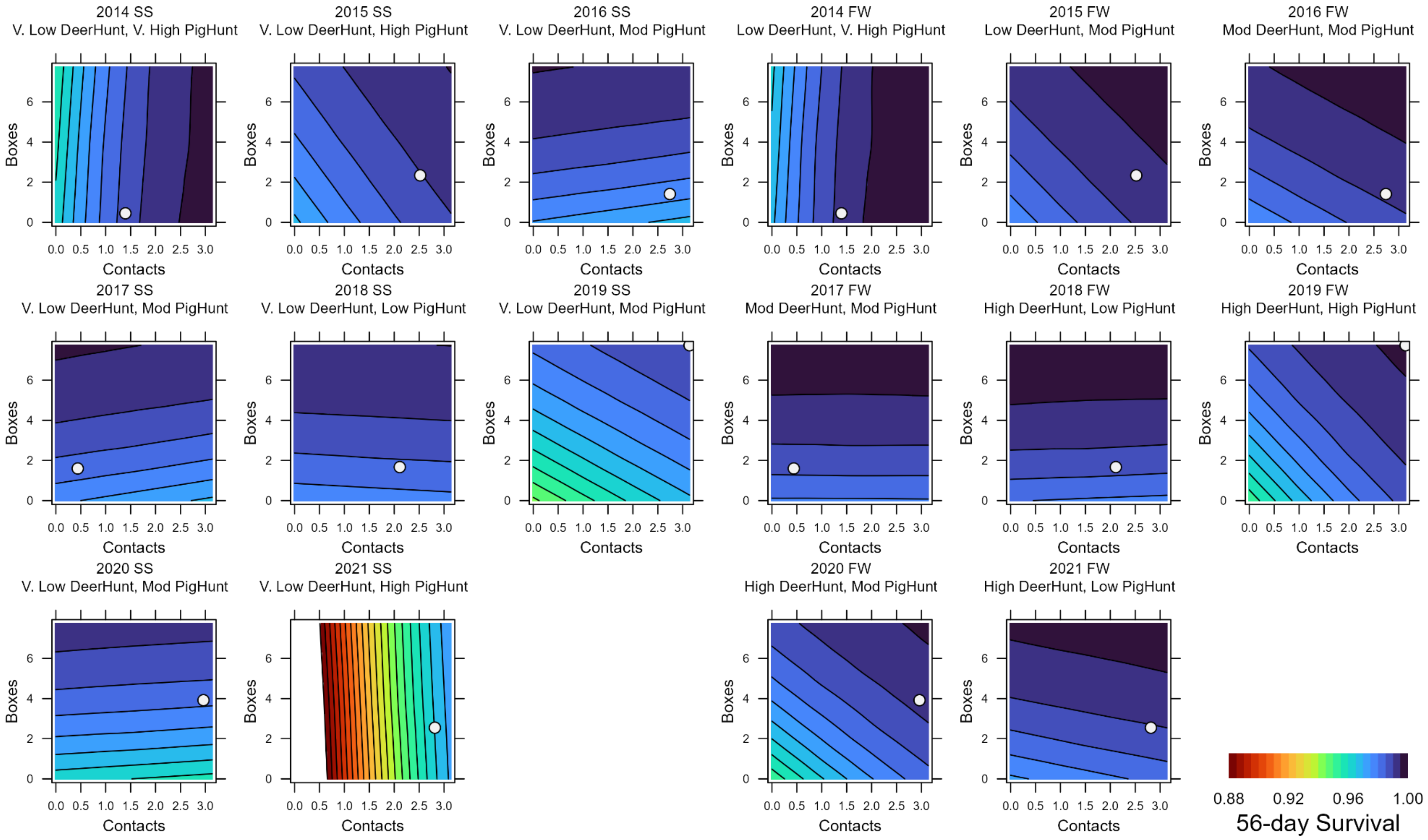

Supplementary Fig. 7. The number of people contacted (*Contacts*) and boxes of ammunition distributed (*Boxes*) increased survival for *Southern* California condors (*Gymnogyps californianus*) across a range of conditions. Plots show predicted 56-day survival for an older adult condor for different combinations of statewide *Contacts* and regional *Boxes* with most model predictors set at their season-specific means (*FallWinter*, *FW* and *SpringSummer*, *SS*). For *FallWinter*, we set *DeerHunt* at its mean for the peak deer hunting months of Oct and Nov. Observed outreach effort (*Contacts* and *Boxes*) for the specific year are plotted (white dot). See Supplementary Note 1 for additional details.

Supplementary Table 1. Variables used in analyses of California condors (*Gymnogyps californianus*). Definitions and descriptions of variables used in models to assess and quantify blood lead levels, carcass contamination rates, survival rates, and reproductive rates for California condors in California and Mexico.

**Variable**	**Definition**
AfterFirstYear	Individuals after their first year after fledging, or age 1.5 (one year after fledging) or older. Individual time-varying covariate. Binary variable.
Age(*i*)	Individual of age(*i*). For example, Age5 is individual 5 years post-fledge, or age 5.5 to 6.5. Individual time-varying covariate. Binary variable.
Ageto(*i*)	Age of individual until Age(i), when age effect plateaus. For example, Ageto9 increments an individual's annual age to age 9, after which variable continues to assign age as 9. Individual time-varying covariate. Integer variable.
Baja (*BACA*)	California condor flock located in Baja California, Mexico (Fig. 1, Supplementary Fig. 1).
Bioyear	Annual time increment, beginning Sep 1 and ending Aug 31 of the following year. For example, *Bioyear* 2008 runs Sep 1, 2008 – Aug 31, 2009. Corresponds to biological year for condors, starting with fledging, which occurs approximately Sep 1.
BloodLead	Blood lead concentrations, reported in µg/dL, as part of biannual health checks (see Methods: Natural history and management relevant to data and Methods: Condor blood lead levels).
Boxes	Number of boxes of nonlead ammunition distributed annually (in 100s) in counties heavily used for foraging by condors, a flock-specific metric. Box distribution started in 2012 for counties heavily used by *Central* and in 2014 for counties heavily used by *Southern*. *Boxes* was summarized over calendar year and used to predict *BloodLead* in the same *Bioyear*, which begins in Sep of the calendar year.
Breeder	Female that has successfully fledged a chick any time in the past.
Central (*CECA)*	California condor flock located in central California (Fig. 1, Supplementary Fig. 1).

Variable	Definition
Coastal	The proportion of all observations of an individual in the previous 180 days that were visual observations in the coastal region, corrected for tracked free-flying days. Individual time-varying covariate. Continuous variable bounded at [0, 1].
Contacts	Number of people contacted annually statewide through educational outreach activities (in 1000s), and thus the number of people who had received information about nonlead ammunition, excluding those receiving boxes of nonlead ammunition. *Contacts* occurred over a longer time frame than *Boxes* (Supplementary Fig. 2), but we limited our analysis to years in which *Boxes* were also distributed. *Contacts* was summarized over calendar year and used to predict *BloodLead* in the same *Bioyear*, which begins in Sep of the calendar year.
DeerHunt	Deer hunting, indexed as the sum of successful tags reported to California Department of Fish and Wildlife in the month of and month prior to blood lead level testing, rescaled by dividing by 1000. For each flock, we summed tags in counties with high foraging use, or, specifically, Monterey, San Benito, and San Luis Obispo for *Central* and Kern and Ventura for *Southern.* Deer harvest prior to 2015 was corrected for more stringent reporting requirements starting 2014. See Methods: Hunting and culling levels and Supplementary Fig. 2 for additional details.<br>• *DeerHunt* is the entire time period of analysis (*Bioyear* 1996 – 2022).<br>• *DeerHunt:Post2008Ban* is DeerHunt in the time period after the 2008 lead ban (*Bioyear* 2008 – 2022).<br>• *DeerHunt:Post2019Ban* is DeerHunt in the time period after the 2019 lead ban (*Bioyear* 2019 – 2022).
EarlyBreedingYrs	First 7 years that breeding age females were present in a flock. Early time period when reproductive rates were lower. Time-varying covariate. Binary variable.
FailedBreeder (FB)	Female breeder that failed to fledge a chick in the previous year.
FallWinter (FW)	Timing within the year: *FallWinter* (FW) season, defined as September – February. Used to test for lead-exposure patterns separate from tag-reported hunting, such as wild pig culling and ground squirrel shooting.
First2Years	Individuals in their first 2 years after fledging, or age 0.5 (age at fledging) to age 2.5. Individual time-varying covariate. Binary variable.
FirstYear	Individuals in their first year after fledging, or age 0.5 (age at fledging) to age 1.5. Individual time-varying covariate. Binary variable.

Variable	Definition
FreeFly	Proportion of days in the previous 180 days that the bird was free-flying (vs. captive in flight pens or zoo facilities). Individual time-varying covariate. Continuous variable bounded at [0, 1].
ID	Individual ID assigned to all condors.
Int	Intercept
Juvenile (JUV)	Individuals 1 to 6 years post-fledge, or age 1.5 to 6.5. Individual time-varying covariate. Binary variable.
NonBreeder (NB)	Female of breeding age that has never fledged a chick in the past (unrecruited breeder).
OlderAdult (OA)	Individuals 10 years post-fledge or older, or 10.5 and older. Individual time-varying covariate. Binary variable.
PigCull	Pig culling, indexed the same way as deer hunting, based on counties of high ground use by condors (see *DeerHunt*), and rescaled by dividing by 100. Due to changes in reporting requirements, reliable data were only available for 2017 onward. Thus, we were unable to use this variable in analyses of predictors of condor lead exposure. However, we explored the relationship between *PigHunt* and *PigCull* (Supplementary Fig. 4).
PigHunt	Pig hunting, indexed the same way as deer hunting, based on counties of high ground use by condor (see *DeerHunt*), and rescaled by dividing by 100. Data were incomplete for *Bioyear* 2011 and thus we interpolated those data as the mean of the corresponding 2010 and 2012 monthly data.<br>• *PigHunt* is the entire time period of analysis (*Bioyear* 1996 – 2022).<br>• *PigHunt:Post2008Ban* is the time period after the 2008 lead ban (*Bioyear* 2008 – 2022).<br>• *PigHunt:Post2019Ban* is the time period after the 2019 lead ban (*Bioyear* 2019 – 2022).
PigHuntCeil	Identical to PigHunt for values <1.0, but values plateau such that all values >1.0 are assigned a value of 1.0. This variable was used for outreach analyses in *Southern* due to high influence of one year with PigHunt > 1.0.<br>• *PigHuntCeil* is the entire time period of analysis (*Bioyear* 1996 – 2022).<br>• *PigHuntCeil:Post2008Ban* is the time period after the 2008 lead ban (*Bioyear* 2008 – 2022).<br>• *PigHuntCeil:Post2019Ban* is the time period after the 2019 lead ban (*Bioyear* 2019 – 2022).
Post2008Ban	Time period starting *Bioyear* 2008, when AB 821, the Ridley-Tree Condor Preservation Act, requiring nonlead ammunition when taking big game and nongame in California condor habitat, was implemented (July 1, 2008). *Bioyears* 2008 – 2022. Binary variable.

Variable	Definition
Post2019Ban	Time period starting *Bioyear* 2019, when AB 8711, a bill modifying AB821 to apply statewide and to the taking of all wildlife, was fully implemented (July 1, 2019). *Bioyears* 2019 – 2022. Binary variable.
PreBreeder (PB)	Female less than age 5, or prior to the age when reproduction has been observed.
Presence	The proportion of all tracking days (days in which field staff attempted to locate condors in a flock) in the previous 180 days that an individual was free-flying and detected by observers (either telemetry signal, visual observation, or camera) after removing proffered feeding observations. This variable documents presence in the release area, broadly defined, independent of proffered feeding observations. Individual time-varying covariate. Continuous variable bounded at [0, 1].
Proffered	The proportion of all tracking days (days in which field staff attempted to locate condors in a flock) in the previous 180 days that an individual was free-flying and observed feeding on a proffered carcass, either visually or on camera. Individual time-varying covariate. Continuous variable bounded at [0, 1].
Released<Yrs	Individual less than *Y* years from initial release to the wild for captive-bred birds. Individual time-varying covariate. Binary variable.
Southern (SOCA)	California condor flock located in southern California (Fig. 1, Supplementary Fig. 1).
$S_{Overall}$	Annual survival of California condors from all causes.
S_{Pb}	Annual survival of California condors from lead-related mortality.
SpringSummer (SS)	Timing within the year: SpringSummer (SS) season, defined as March – August. Used to test for lead-exposure patterns separate from tag-reported hunting, such as wild pig culling and ground squirrel shooting.
Succ	Probability of a female successfully fledging a chick in the current year.
SuccessfulBreeder (SB)	Female breeder that successfully fledged a chick in the previous year.
Targeted	Variable indicating if a blood sample was taken due to suspected lead exposure vs. routine health checks. Individual time-varying covariate. Binary variable.

Variable	Definition
YoungAdult (YA)	Individuals 6 to 10 years post-fledge, or age 6.5 to age 10.5. Individual time-varying covariate. Binary variable.
Yr08on	Equivalent to *Post2008Ban*
Yr19on	Equivalent to *Post2019Ban*
Yr<Yyrs	Time period less than Y years from the start of releases for a flock. Used to discount period of adjustment at each release site when flocks were small. Time-varying covariate. Binary variable.

Supplementary Table 2. Age- and stage-based matrix model for California condors (*Gymnogyps californianus*) – model structure. See Supplementary Table 1 for variable definitions and Supplementary Tables 7-8 for parameter values.

	PB1	PB2	PB3	PB4	PB5	NB6	SB6	NB7	FB7	SB7	NB8	SB8	FB8	NB9on	FB9on	SB9on
**PB1**							Repro			Repro		Repro				Repro
**PB2**	PB1→ PB2															
**PB3**		PB2→ PB3														
**PB4**			PB3→ PB4													
**PB5**				PB4→ PB5												
**NB6**					PB5→ NB6											
**SB6**					PB5→ SB6											
**NB7**						NB6→ NB7										
**FB7**							SB6→ FB7									
**SB7**						NB6→ SB7	SB6→ SB7									
**NB8**								NB7→ NB8								
**SB8**								NB7→ SB8	FB7→ SB8	SB7→ SB8						
**FB8**									FB7→ FB8	SB7→ FB8						
**NB9on**											NB8→ NB9on			NB9on→ NB9on		
**FB9on**												SB8→ FB9on	FB8→ FB9on		FB9on→ FB9on	SB9on→ FB9on
**SB9on**											NB8→ SB9on	SB8→ SB9on	FB8→ SB9on	NB9on→ SB9on	FB9on→ SB9on	SB9on→ SB9on

Transitions were defined as follows:

$Repro = female \cdot S_{FirstYear}$

$PB_i \rightarrow PB_i = S_{AfterFirstYear}$

$PB5 \rightarrow NB6 = S_{AfterFirstYear} \cdot (1 - Succ_{NB5})$

$PB5 \rightarrow SB6 = S_{AfterFirstYear} \cdot Succ_{NB5}$

$NB_i \rightarrow NB_{i+1} = S_{AfterFirstYear} \cdot (1 - Succ_{NBi})$

$NB_i \rightarrow SB_{i+1} = S_{AfterFirstYear} \cdot Succ_{NBi}$

$SB_i \rightarrow FB_{i+1} = S_{AfterFirstYear} \cdot (1 - Succ_{SBi})$

$SB_i \rightarrow SB_{i+1} = S_{AfterFirstYear} \cdot Succ_{SBi}$

$FB_i \rightarrow FB_{i+1} = S_{AfterFirstYear} \cdot (1 - Succ_{FBi})$

$FB_i \rightarrow SB_{i+1} = S_{AfterFirstYear} \cdot Succ_{FBi}$

Supplementary Table 3. Predictors of blood lead levels for California condors (*Gymnogyps californianus*) in California. Coefficients for best model to predict log-transformed blood lead levels using LMMs in R for California condors in (**a**) *Central* and (**b**) *Southern* for a total of 3179 samples of 367 California condors over 27 years. See Supplementary Tables 9-10 for model selection and Supplementary Table 1 for variable definitions.

a. CENTRAL

Fixed effect	Estimate	Std.	df	t	Pr(>|t|)
Intercept	1.963	0.330	1041.839	5.944	<<0.001
Targeted	1.270	0.149	1364.163	8.508	<<0.001
Condor age and time since release					
FirstYear	-0.465	0.142	1342.738	-3.280	0.001
YoungAdult	0.118	0.069	1357.662	1.695	0.090
Released<2yrs	-0.366	0.078	1370.622	-4.697	<<0.001
Condor behaviors					
FreeFly	1.207	0.291	1377.533	4.142	<<0.001
Presence	-0.358	0.198	1234.399	-1.814	0.070
Proffered	-1.500	0.339	1380.540	-4.424	<<0.001
Coastal	-1.076	0.155	1185.767	-6.946	<<0.001
Lead sources and timing					
Post2019Ban	-0.711	0.381	246.088	-1.865	0.063
SpringSummer	-0.151	0.082	775.085	-1.840	0.066
DeerHunt	0.319	0.083	1257.670	3.838	<0.001
PigHunt	0.109	0.044	125.282	2.481	0.014
PigHunt: Post2008Ban	0.232	0.049	82.335	4.721	<<0.001
PigHunt: Post2019Ban	0.434	0.131	486.758	3.313	0.001
**Random effect**					
Groups	Variance				
ID	0.133				
Bioyear	0.049				
Residual	0.930				
Number of obs:	1421				
ID:	188				
Bioyear	26				
**R-squared statistics**					
Marginal R^2	0.262				
Conditional R^2	0.383				

b. SOUTHERN

Fixed effect	Estimate	Std.	df	t	Pr(>|t|)
Intercept	2.874	0.273	640.708	10.518	<<0.001
Targeted	0.614	0.105	1679.813	5.853	<<0.001
Condor age and time since release					
FirstYear	-0.250	0.123	1685.557	-2.024	0.043
YoungAdult	0.089	0.055	1732.915	1.626	0.104
Released<2yrs	-0.311	0.063	1528.755	-4.929	<<0.001
Condor behaviors					
FreeFly	0.873	0.215	1734.734	4.051	<<0.001
Presence	-0.644	0.152	1200.148	-4.244	<<0.001
Proffered	-3.065	0.451	1276.447	-6.802	<<0.001
Lead sources and timing					
Post2008Ban	-0.328	0.168	42.153	-1.955	0.057
SpringSummer	0.287	0.046	1629.745	6.194	<<0.001
DeerHunt: Post2019Ban	-0.636	0.228	1453.855	-2.790	0.005
PigHunt: Post2019Ban	0.586	0.225	128.375	2.604	0.010
**Random effect**					
Groups	Variance				
ID	0.082				
Bioyear	0.107				
Residual	0.709				
Number of obs:	1758				
ID:	179				
Bioyear	27				
**R-squared statistics**					
Marginal R^2	0.175				
Conditional R^2	0.348				

Supplementary Table 4. Predictors of contaminated meal exposure rates for California condors (*Gymnogyps californianus*) in California. Coefficients for Poisson-Gamma model to predict log-transformed blood lead levels for California condors in (**a**) *Central* and (**b**) *Southern* as well as estimated carcass contamination rates through time. Model is the best model based on LMM model selection (Supplementary Table 3) and was based on the same data, or a total of 3179 samples of 367 California condors over 27 years. See Supplementary Table 1 for variable definitions.

a. CENTRAL

	Coefficient Estimate			Mean covariate value by time period		
**Parameter**	**Lower 95**	**Median**	**Upper 95**	**1996 - 2007**	**2008 - 2018**	**2019 - 2022**
**Poisson**						
Intercept	3.470	4.256	5.174	1	1	1
Targeted	0.136	0.517	0.880	0.066	0.030	0.031
Condor age and time since release						
FirstYear	-0.715	-0.453	-0.206	0.051	0.035	0.082
YoungAdult	-0.019	0.091	0.198	0.136	0.234	0.204
Released<2yrs	-0.377	-0.244	-0.116	0.425	0.165	0.153
Condor behaviors						
FreeFly	0.257	0.757	1.392	0.939	0.940	0.962
Presence	-0.468	-0.196	0.060	0.609	0.527	0.406
Proffered	-1.730	-1.152	-0.613	0.209	0.135	0.187
Coastalness	-1.022	-0.806	-0.566	0.297	0.274	0.269
Lead sources and timing						
Post2019Ban	-0.792	-0.228	0.219	0	0	1
SpringSummer	-0.152	-0.035	0.087	0.579	0.483	0.459
DeerHunt	0.130	0.370	0.610	0.124	0.151	0.107
PigHunt	-0.164	0.245	0.643	0.471	0.287	0.414
PigHunt:Post2008Ban	0.805	1.168	1.551	0.000	0.287	0.414
PigHunt:Post2019Ban	0.259	1.277	2.599	0.000	0.000	0.414
**Gamma mean effects and shape parameter**						
(Intercept)	-1.810	-1.247	0.125	1	1	1
Targeted	0.162	0.613	1.058	0.066	0.030	0.031
Shape	0.003	0.005	0.020			

**Mean estimates per time period**	**1996 - 2007**	**2008 - 2018**	**2019 - 2022**
Lead per carcass (µg/dL)	0.30	0.29	0.29
Contaminated carcasses encountered	83.8	136.2	208.5
Carcasses encountered contributing >10 µg/dL to mean blood sample	0.6	0.9	1.4
Annual carcasses encountered contributing >10 µg/dL	3.8	6.1	9.4

b. SOUTHERN

	Coefficient Estimate			Mean covariate value by time period		
**Fixed effect**	**Lower 95**	**Median**	**Upper 95**	**1996 - 2007**	**2008 - 2018**	**2019 - 2022**
**Poisson**						
Intercept	4.042	4.986	5.580	1	1	1
Targeted	-0.370	-0.086	0.193	0.062	0.054	0.000
Condor age and time since release						
FirstYear	-0.330	-0.101	0.114	0.059	0.032	0.013
YoungAdult	-0.011	0.082	0.171	0.244	0.193	0.204
Released<2yrs	-0.314	-0.206	-0.097	0.267	0.190	0.105
Condor behaviors						
FreeFly	0.184	0.548	0.969	0.942	0.932	0.958
Presence	-0.989	-0.800	-0.599	0.648	0.503	0.172
Proffered	-2.065	-1.416	-0.789	0.199	0.052	0.026
Lead sources and timing						
Post2008Ban	-0.228	-0.106	0.019	0	1	1
SpringSummer	0.096	0.178	0.257	0.579	0.507	0.184
DeerHunt:Post2019Ban	-0.514	-0.268	-0.022	0.000	0.000	0.371
PigHunt:Post2019Ban	-0.266	0.295	0.852	0.000	0.000	0.211
**Gamma mean effects and shape parameter**						
(Intercept)	-1.952	-1.475	-0.396	1	1	1
Targeted	0.457	0.817	1.207	0.062	0.054	0.000
Shape	0.004	0.007	0.019			

**Mean estimates per time period**	**1996 - 2007**	**2008 - 2018**	**2019 - 2022**
Lead per carcass (µg/dL)	0.24	0.24	0.23
Contaminated carcasses encountered	116.7	144.5	184.9
Carcasses encountered contributing >10 µg/dL to mean blood sample	0.8	0.9	1.1
Annual carcasses encountered contributing >10 µg/dL	4.9	6.0	7.4

Supplementary Table 5. Predictors of blood lead levels for California condors (*Gymnogyps californianus*) in California when outreach was occurring. Coefficients for best model to predict log-transformed blood lead levels for California condors including nonlead outreach metrics using LMMs in (**a**) *Central* and (**b**) *Southern* for a total of 1360 samples of 276 California condors over 10 years. These analyses were restricted to time period when outreach occurred, or *Bioyear* 2012 – 2021 for *Central* and 2014 – 2021 for *Southern*. See Supplementary Tables 11-12 for model selection and Supplementary Table 1 for variable definitions.

a. CENTRAL

**Fixed effect**	**Coefficient Estimate**	**Std. Error**	**df**	**t value**	**Pr(>|t|)**
Intercept	1.303	0.701	59.164	1.859	0.068
Targeted	1.711	0.421	636.776	4.065	<<0.001
Condor age and time since release					
FirstYear	-0.625	0.213	673.454	-2.936	0.003
YoungAdult	0.248	0.102	678.851	2.438	0.015
Released<2yrs	-0.288	0.120	673.254	-2.398	0.017
Condor behaviors					
FreeFly	1.237	0.484	665.832	2.558	0.011
Proffered	-1.869	0.491	545.385	-3.807	<0.001
Coastal	-0.569	0.191	541.779	-2.983	0.003
Lead sources and timing and outreach					
SpringSummer	0.371	0.147	42.532	2.522	0.016
DeerHunt	1.946	0.510	545.271	3.819	<0.001
PigHunt	0.384	0.198	24.889	1.941	0.064
PigHunt:Post2019Ban	0.293	0.054	10.468	5.400	<0.001
Boxes	-0.004	0.019	7.676	-0.203	0.845
Contacts	0.430	0.223	31.609	1.926	0.063
Boxes:DeerHunt	-0.088	0.036	614.015	-2.417	0.016
Contacts:DeerHunt	-0.442	0.152	471.234	-2.916	0.004
Contacts:PigHunt	-0.172	0.081	38.987	-2.136	0.039
**Random effect**	**Variance**				
ID	0.17466				
Bioyear	0.00295				
Residual	0.93773				
Number of obs:	699				
ID	152				
Bioyear	10				
**R-squared statistics**					
Marginal R^2	0.182				
Conditional R^2	0.312				

b. SOUTHERN

**Fixed effect**	**Coefficient Estimate**	**Std. Error**	**df**	**t value**	**Pr(>|t|)**
Intercept	1.251	0.770	112.562	1.625	0.107
Condor age and time since release					
FirstYear	-0.329	0.225	620.470	-1.465	0.144
YoungAdult	0.220	0.082	608.119	2.702	0.007
Released<2yrs	-0.333	0.103	592.961	-3.223	0.001
Condor behaviors					
FreeFly	2.325	0.611	643.697	3.807	<0.001
Proffered	-5.964	0.739	553.558	-8.067	<<0.001
Lead sources and timing and outreach					
Post2019Ban	-1.601	0.411	27.422	-3.892	0.001
SpringSummer	0.473	0.099	238.267	4.780	<<0.001
DeerHunt	0.838	0.601	567.943	1.395	0.164
PigHuntCeil	0.704	0.747	45.500	0.942	0.351
PigHuntCeil:Post2019Ban	3.493	0.597	132.173	5.849	<<0.001
Boxes	-0.418	0.201	86.273	-2.076	0.041
Contacts	0.835	0.249	36.887	3.353	0.002
Contacts:DeerHunt	-0.459	0.217	520.726	-2.118	0.035
Boxes:PigHuntCeil	0.437	0.262	128.831	1.669	0.098
Contacts:PigHuntCeil	-1.570	0.407	86.638	-3.861	<0.001
**Random effect**	**Variance**				
ID	0.098				
Bioyear	0.018				
Residual	0.560				
Number of obs:	661				
ID	124				
Bioyear	8				
**R-squared statistics**					
Marginal R^2	0.316				
Conditional R^2	0.433				

Supplementary Table 6. Predictors of survival for California condors (*Gymnogyps californianus*) in California. Predictors of (**a**) $S_{Overall}$ and (**b**) S_{Pb} for California condors in *Central* (CECA) and *Southern* (SOCA) using known fate models based on near-daily records for all free-flying condors for *Bioyear* 1996 through 2022, or 1452 annual records for 226 condors in *Central* and 1428 annual records for 226 condors in *Southern*. See Supplementary Tables 13-14 for model selection and Supplementary Table 1 for variable definitions.

**a. $S_{Overall}$**					**b. S_{Pb}**				
**Effect**	**Coefficient Estimate**	**Std. Error**	**Lower 95**	**Upper 95**	**Effect**	**Coefficient Estimate**	**Std. Error**	**Lower 95**	**Upper 95**
Intercept	1.933	0.184	1.572	2.295	Intercept	3.269	0.216	2.845	3.693
Site and early effects									
CECA	1.171	0.351	0.483	1.858					
Yr<6yrs	-1.523	0.312	-2.134	-0.911					
CECA:Yr<6Yrs	3.595	1.115	1.409	5.781					
Condor age and time since release					*Condor age and time since release*				
FirstYear	-0.602	0.261	-1.114	-0.090	First2Years	1.194	0.479	0.256	2.132
YoungAdult	-0.251	0.167	-0.579	0.077	YoungAdult	-0.459	0.233	-0.916	-0.002
Released<1Yr	-0.947	0.194	-1.327	-0.567					
Condor behaviors					*Condor behaviors*				
Proffered	2.528	0.672	1.210	3.846	Proffered	3.635	1.265	1.155	6.116
SOCA:Presence	1.029	0.386	0.273	1.785					
					CECA:Coastal	1.224	0.432	0.378	2.071
Lead sources and timing and outreach					*Lead sources and timing and outreach*				
CECA:PigHunt	-0.277	0.121	-0.514	-0.040					
					CECA:PigHunt: Post2008Ban	-0.447	0.123	-0.688	-0.206
CECA:PigHunt: Post2019Ban	-0.218	0.075	-0.366	-0.071	CECA:PigHunt: Post2019Ban	-0.273	0.110	-0.488	-0.058
					SOCA:DeerHunt: Post2008Ban	4.069	1.895	0.354	7.784
SOCA:DeerHunt: Post2019Ban	2.061	1.131	-0.155	4.277					

Supplementary Table 7. Age- and stage-based matrix model for *Central* and *Southern* – parameter values. Deterministic lambda = 0.971. See Supplementary Table 1 for variable definitions and Supplementary Table 2 for transition equations. Note this matrix excludes released individuals.

	PB1	PB2	PB3	PB4	PB5	NB6	SB6	NB7	FB7	SB7	NB8	FB8	SB8	NB9on	FB9on	SB9on
**PB1**	0	0	0	0	0	0	0.42	0	0	0.42	0	0	0.42	0	0	0.42
**PB2**	0.91	0	0	0	0	0	0	0	0	0	0	0	0	0	0	0
**PB3**	0	0.91	0	0	0	0	0	0	0	0	0	0	0	0	0	0
**PB4**	0	0	0.91	0	0	0	0	0	0	0	0	0	0	0	0	0
**PB5**	0	0	0	0.91	0	0	0	0	0	0	0	0	0	0	0	0
**NB6**	0	0	0	0	0.90	0	0	0	0	0	0	0	0	0	0	0
**SB6**	0	0	0	0	0.01	0	0	0	0	0	0	0	0	0	0	0
**NB7**	0	0	0	0	0	0.82	0	0	0	0	0	0	0	0	0	0
**FB7**	0	0	0	0	0	0	0.89	0	0	0	0	0	0	0	0	0
**SB7**	0	0	0	0	0	0.09	0.02	0	0	0	0	0	0	0	0	0
**NB8**	0	0	0	0	0	0	0	0.79	0	0	0	0	0	0	0	0
**FB8**	0	0	0	0	0	0	0	0	0.73	0.88	0	0	0	0	0	0
**SB8**	0	0	0	0	0	0	0	0.11	0.18	0.03	0	0	0	0	0	0
**NB9on**	0	0	0	0	0	0	0	0	0	0	0.76	0	0	0.72	0	0
**FB9on**	0	0	0	0	0	0	0	0	0	0	0	0.63	0.85	0	0.63	0.85
**SB9on**	0	0	0	0	0	0	0	0	0	0	0.15	0.28	0.06	0.19	0.28	0.06

Supplementary Table 8. Age- and stage-based matrix for *Baja* – parameter values. Deterministic lambda = 1.017. See Supplementary Table 1 for variable definitions and Supplementary Table 2 for transition equations. Note this matrix excludes released individuals.

	PB1	PB2	PB3	PB4	PB5	NB6	SB6	NB7	FB7	SB7	NB8	FB8	SB8	NB9on	FB9on	SB9on
**PB1**	0	0	0	0	0	0	0.45	0	0	0.45	0	0	0.45	0	0	0.45
**PB2**	0.95	0	0	0	0	0	0	0	0	0	0	0	0	0	0	0
**PB3**	0	0.95	0	0	0	0	0	0	0	0	0	0	0	0	0	0
**PB4**	0	0	0.95	0	0	0	0	0	0	0	0	0	0	0	0	0
**PB5**	0	0	0	0.95	0	0	0	0	0	0	0	0	0	0	0	0
**NB6**	0	0	0	0	0.94	0	0	0	0	0	0	0	0	0	0	0
**SB6**	0	0	0	0	0.01	0	0	0	0	0	0	0	0	0	0	0
**NB7**	0	0	0	0	0	0.86	0	0	0	0	0	0	0	0	0	0
**FB7**	0	0	0	0	0	0	0.93	0	0	0	0	0	0	0	0	0
**SB7**	0	0	0	0	0	0.09	0.02	0	0	0	0	0	0	0	0	0
**NB8**	0	0	0	0	0	0	0	0.83	0	0	0	0	0	0	0	0
**FB8**	0	0	0	0	0	0	0	0	0.76	0.92	0	0	0	0	0	0
**SB8**	0	0	0	0	0	0	0	0.12	0.19	0.03	0	0	0	0	0	0
**NB9on**	0	0	0	0	0	0	0	0	0	0	0.79	0	0	0.75	0	0
**FB9on**	0	0	0	0	0	0	0	0	0	0	0	0.71	0.91	0	0.65	0.89
**SB9on**	0	0	0	0	0	0	0	0	0	0	0.16	0.24	0.04	0.20	0.30	0.06

Supplementary Table 9. Model selection table for predictors of lead exposure for California condors (*Gymnogyps californianus*) in *Central*. Shown are coefficient and AICc values for the top set (delta AICc ≤ 2) of LMMs to predict log-transformed *BloodLead*. Shaded models have higher ranked models nested within them and thus contain parameters likely to be uninformative. See Supplementary Fig. 3 for measures of variable importance. Data consisted of 1421 *BloodLead* samples for 188 condors over 26 years. All models also included *Bioyear* and *ID* as random effects. See Supplementary Table 1 for variable definitions. Model in Supplementary Table 3a is the top-ranked model here; coefficient values may differ slightly because model selection was performed using REML = false, while final models were fit using REML = true.

Int	Targeted	First Year	Young Adult	Released <2yrs	FreeFly	Presence	Proffered	Coastal	Post 2008Ban	Post 2019Ban	Spring Summer	Deer Hunt	Pig Hunt	Post 2008Ban: DeerHunt	Post 2019Ban: DeerHunt	Post 2008Ban: PigHunt	Post 2019Ban: PigHunt	df	logLik	AICc	delta AICc
1.97	1.27	-0.47	0.12	-0.37	1.20	-0.37	-1.51	-1.07		-0.68	-0.15	0.32	0.11			0.23	0.42	18	-2032.11	4100.71	0.00
1.99	1.28	-0.47	0.12	-0.37	1.19	-0.37	-1.49	-1.08		-0.67	-0.16	0.47	0.10	-0.19		0.24	0.42	19	-2031.36	4101.27	0.56
1.99	1.26	-0.49		-0.39	1.21	-0.34	-1.53	-1.06		-0.70	-0.16	0.31	0.11			0.23	0.43	17	-2033.54	4101.52	0.81
1.89	1.26	-0.47	0.12	-0.36	1.19	-0.35	-1.54	-1.04			-0.12	0.37	0.12		-0.29	0.24	0.22	18	-2032.66	4101.81	1.10
1.95	1.26	-0.47	0.12	-0.36	1.21	-0.37	-1.49	-1.07		-0.56	-0.14	0.35	0.11		-0.19	0.23	0.39	19	-2031.69	4101.92	1.21
1.90	1.27	-0.46	0.12	-0.36	1.18	-0.32	-1.59	-1.03			-0.13	0.33	0.12			0.24	0.21	17	-2033.77	4101.99	1.28
2.02	1.27	-0.49		-0.40	1.19	-0.35	-1.52	-1.06		-0.68	-0.17	0.46	0.10	-0.20		0.24	0.42	18	-2032.76	4102.01	1.30
2.03	1.27	-0.49	0.12	-0.37	1.18	-0.39	-1.56	-1.09		-0.60		0.40	0.08			0.21	0.41	17	-2033.79	4102.03	1.32
1.95	1.27	-0.49	0.13	-0.36	1.18	-0.37	-1.57	-1.06				0.44	0.09		-0.31	0.22	0.23	17	-2033.80	4102.03	1.32
1.74	1.27	-0.42	0.11	-0.36	1.18		-1.55	-0.97		-0.61	-0.16	0.31	0.11			0.25	0.40	17	-2033.83	4102.10	1.39
1.93	1.28	-0.47	0.12	-0.37	1.16	-0.33	-1.57	-1.03			-0.14	0.48	0.11	-0.20		0.25	0.21	18	-2032.97	4102.43	1.72
1.78	1.26	-0.44		-0.38	1.18		-1.57	-0.97		-0.63	-0.17	0.29	0.11			0.26	0.41	16	-2035.07	4102.53	1.82
1.96	1.27	-0.48	0.13	-0.37	1.16	-0.35	-1.63	-1.05				0.40	0.09			0.21	0.22	16	-2035.10	4102.58	1.87
1.70	1.27	-0.42	0.11	-0.35	1.16		-1.62	-0.94			-0.14	0.31	0.12			0.26	0.21	16	-2035.15	4102.68	1.97

Supplementary Table 10. Model selection table for predictors of lead exposure for California condors (*Gymnogyps californianus*) in *Southern*. Shown are coefficient and AICc values for the top set (delta AICc ≤ 2) of LMMs to predict log-transformed *BloodLead*. Shaded models have higher ranked models nested within them and thus contain parameters likely to be uninformative. See Supplementary Fig. 3 for measures of variable importance. Data consisted of 1758 *BloodLead* samples for 179 condors over 27 years. All models also included *Bioyear* and *ID* as random effects. See Supplementary Table 1 for variable definitions. Model in Supplementary Table 3b is the top-ranked model here. Coefficient values may differ slightly because model selection was performed using REML = false, while final models were fit using REML = true.

Int	Targeted	First Year	Young Adult	Released <2yrs	FreeFly	Presence	Proffered	Post 2008Ban	Post 2019Ban	Spring Summer	Deer Hunt	Pig Hunt	Post 2008Ban: DeerHunt	Post 2019Ban: DeerHunt	Post 2008Ban: PigHunt	Post 2019Ban: PigHunt	df	logLik	AICc	delta AICc
2.87	0.61	-0.25	0.09	-0.31	0.88	-0.65	-3.04	-0.32		0.29				-0.64		0.57	15	-2273.36	4576.99	0.00
2.86	0.61	-0.25	0.09	-0.31	0.88	-0.67	-3.04	-0.35		0.32			0.21	-0.80		0.58	16	-2272.60	4577.51	0.52
2.91	0.62	-0.26		-0.34	0.88	-0.66	-3.08	-0.34		0.29				-0.64		0.57	14	-2274.69	4577.61	0.62
2.82	0.61	-0.25	0.09	-0.32	0.89	-0.68	-3.00	-0.31		0.33	0.19			-0.78		0.57	16	-2272.71	4577.74	0.74
2.89	0.61	-0.26		-0.34	0.89	-0.68	-3.08	-0.36		0.33			0.21	-0.80		0.57	15	-2273.92	4578.12	1.12
2.86	0.62	-0.26		-0.34	0.89	-0.69	-3.03	-0.33		0.33	0.19			-0.78		0.56	15	-2274.04	4578.36	1.36
2.89	0.62	-0.25	0.09	-0.31	0.88	-0.66	-3.01	-0.33		0.29		-0.03		-0.64		0.59	16	-2273.18	4578.68	1.69
2.87	0.61	-0.25	0.09	-0.31	0.88	-0.66	-3.04	-0.34		0.29				-0.63	0.03	0.56	16	-2273.29	4578.88	1.89
2.61	0.62	-0.23	0.09	-0.32	0.89	-0.63	-2.66			0.29				-0.66		0.49	14	-2275.33	4578.91	1.92

Supplementary Table 11. Model selection table for predictors of lead exposure for California condors (*Gymnogyps californianus*) in *Central* including outreach metrics. Shown are coefficient and AICc values for the top set (delta AICc ≤ 2) of LMMs to predict log-transformed *BloodLead*. Shaded models have higher ranked models nested within them and thus contain parameters likely to be uninformative. See Supplementary Fig. 3 for measures of variable importance. Data consisted of 699 *BloodLead* samples for 152 condors for *Bioyear* 2012 - 2021. All models also included *Bioyear* and *ID* as random effects. See Supplementary Table 1 for variable definitions. Model in Supplementary Table 5a is the top-ranked model here; coefficient values may differ slightly because model selection was performed using REML = false, while final models were fit using REML = true.

Int	Targeted	First Year	Young Adult	Released <2yrs	Free Fly	Proff-ered	Coastal-ness	Post 2019Ban	Spring Summer	Deer Hunt	Pig Hunt	Boxes	Contacts	Post 2019Ban: DeerHunt	Post 2019Ban: PigHunt	Boxes: DeerHunt	Contacts: DeerHunt	Boxes: PigHunt	Contacts: PigHunt	df	logLik	AICc	delta AICc
1.21	1.70	-0.63	0.25	-0.28	1.23	-1.87	-0.57		0.38	1.98	0.41	0.00	0.47		0.30	-0.09	-0.45		-0.19	20	-1004.48	2050.19	0.00
2.32	1.76	-0.63	0.25	-0.29	1.29	-1.89	-0.60		0.39	1.58		-0.01	-0.03		0.26	-0.08	-0.30			18	-1007.13	2051.28	1.08
1.12	1.71	-0.62	0.24	-0.28	1.22	-1.77	-0.59	-0.38	0.34	1.87	0.47	-0.01	0.55		0.43	-0.08	-0.45		-0.22	21	-1004.14	2051.65	1.46
1.59	1.73	-0.63	0.25	-0.29	1.21	-1.90	-0.58		0.36	1.80	0.30	-0.04	0.46		0.29	-0.07	-0.44	0.01	-0.18	21	-1004.20	2051.76	1.57
1.22	1.70	-0.63	0.25	-0.29	1.21	-1.92	-0.55		0.38	2.13	0.41	0.00	0.48	0.19	0.29	-0.10	-0.50		-0.19	21	-1004.30	2051.97	1.78
2.21	1.77	-0.65	0.26	-0.30	1.14	-2.02	-0.60		0.30	1.14	0.16	-0.11	0.41		0.28		-0.38	0.03	-0.17	20	-1005.43	2052.10	1.91

Supplementary Table 12. Model selection table for predictors of lead exposure for California condors (*Gymnogyps californianus*) in *Southern* including outreach metrics. Shown are coefficient and AICc values for the top set (delta AICc ≤ 2) of LMMs to predict log-transformed *BloodLead*. Shaded models have higher ranked models nested within them and thus contain parameters likely to be uninformative. See Supplementary Fig. 3 for measures of variable importance. Data consisted of 661 *BloodLead* samples for 124 condors for *Bioyear* 2014 - 2021. All models also included *Bioyear* and *ID* as random effects. See Supplementary Table 1 for variable definitions. Model in Table Supplementary 5b is the top-ranked model here; coefficient values may differ slightly because model selection was performed using REML = false, while final models were fit using REML = true.

Int	First Year	Young Adult	Released <2yrs	FreeFly	Proffered	Post 2019Ban	Spring Summer	Deer Hunt	Pig Hunt Ceil	Boxes	Contacts	Post 2019Ban: DeerHunt	Post 2019Ban: PigHunt Ceil	Boxes: DeerHunt	Contacts: DeerHunt	Boxes: PigHunt Ceil	Contacts: PigHunt Ceil	df	logLik	AICc	delta AICc
1.75	-0.35	0.23	-0.32	2.21	-6.07	-1.46	0.44	0.70	0.27	-0.60	0.86		3.34		-0.47	0.67	-1.60	19	-779.26	1597.71	0.00
1.69		0.25	-0.31	2.22	-6.03	-1.47	0.43	0.68	0.30	-0.58	0.86		3.35		-0.46	0.64	-1.59	18	-780.54	1598.15	0.43
1.17		0.23	-0.32	2.29	-5.92	-2.01	0.42	0.33	0.77	-0.14	0.78		4.02	0.22	-0.54		-1.42	18	-780.59	1598.25	0.54
1.71	-0.36	0.23	-0.33	2.20	-6.07	-1.81	0.40	0.74	0.38	-0.58	0.96	0.56	3.76		-0.64	0.63	-1.72	20	-778.53	1598.37	0.66
1.23	-0.31	0.22	-0.33	2.27	-5.94	-2.01	0.43	0.33	0.73	-0.14	0.78		4.02	0.22	-0.54		-1.40	19	-779.62	1598.43	0.72
1.66		0.24	-0.31	2.21	-6.03	-1.81	0.39	0.72	0.40	-0.56	0.95	0.55	3.77		-0.62	0.61	-1.71	19	-779.83	1598.84	1.12
1.66	-0.34	0.23	-0.32	2.23	-6.07	-1.62	0.43	0.52	0.35	-0.49	0.86		3.55	0.11	-0.53	0.49	-1.56	20	-778.91	1599.14	1.43
1.53		0.24	-0.31	2.25	-6.02	-1.67	0.42	0.50	0.44	-0.43	0.85		3.61	0.13	-0.53	0.41	-1.54	19	-780.10	1599.39	1.68

Supplementary Table 13. Model selection table for predictors of overall survival ($S_{Overall}$) for California condors (*Gymnogyps californianus*). Shown are model forms and AICc values for the top 20 known fate models to predict survival overall. Shaded models have higher ranked models nested within and thus contain parameters likely to be uninformative. Data consisted of near-daily records for all free-flying condors in the *Central* (CECA) and *Southern* (SOCA) flocks in California from *Bioyear* 1996 through 2022, or 1452 annual records for 226 condors and 117 deaths in *Central* and 1428 annual records for 226 condors and 134 deaths in *Southern*. Model in Supplementary Table 6a is the top-ranked model here. See Supplementary Table 1 for variable definitions. Other abbreviations: K is number of parameters.

					Yrto7	Coastal	Proffered			Presence		Post08Ban		Post19Ban		DeerHunt		PigHunt		Post08Ban: DeerHunt		Post19Ban: DeerHunt		Post08Ban: PigHunt		Post19Ban: PigHunt							
First Year	Rel <1Yr	Young Adult	CECA	Yrto7	CECA	CECA	--	CECA	SOCA	CECA	SOCA	CECA	SOCA	CECA	SOCA	CECA	SOCA	CECA	SOCA	CECA	SOCA	CECA	SOCA	CECA	SOCA	CECA	SOCA	K	AICc	delta AICc	AICc weight	Deviance	Cumul. weight
X	X	X	X	X	X		X				X							X					X			X		12	2527.85	0.00	0.14	2503.83	0.14
X	X	X	X	X	X		X				X			X				X					X					12	2527.98	0.13	0.13	2503.96	0.27
X	X		X	X	X		X				X							X					X			X		11	2528.03	0.18	0.13	2506.01	0.40
X	X	X	X	X	X		X				X							X		X			X			X		13	2528.67	0.82	0.09	2502.64	0.50
X	X	X	X	X	X		X				X							X	X				X			X		13	2528.80	0.95	0.09	2502.78	0.58
X	X	X	X	X	X		X				X							X		X		X	X			X		14	2529.45	1.60	0.06	2501.42	0.65
X	X	X	X	X	X		X				X							X					X	X		X		13	2529.78	1.93	0.05	2503.75	0.70
X	X	X	X	X	X		X				X							X					X			X	X	13	2529.79	1.95	0.05	2503.77	0.76
X	X	X	X	X	X		X				X							X				X	X			X		13	2529.83	1.99	0.05	2503.81	0.81
X	X	X	X	X	X		X				X							X								X		11	2529.96	2.12	0.05	2507.95	0.86
X	X	X	X	X	X		X				X							X	X	X		X	X			X		15	2530.41	2.56	0.04	2500.38	0.90
X	X	X	X	X	X		X				X				X			X								X		12	2531.13	3.28	0.03	2507.11	0.92
X	X	X	X	X	X		X				X						X	X								X		12	2531.83	3.98	0.02	2507.81	0.94
X	X	X	X	X	X		X				X							X	X		X					X		13	2532.11	4.27	0.02	2506.09	0.96
X	X	X	X	X	X		X				X					X		X	X	X		X	X			X		16	2532.14	4.30	0.02	2500.11	0.98
X	X	X	X	X	X		X				X							X					X	X				12	2533.09	5.25	0.01	2509.07	0.99
X	X	X	X	X	X		X				X	X				X		X	X	X		X	X			X		17	2533.72	5.87	0.01	2499.68	0.99
X	X	X	X	X	X			X	X		X	X				X		X	X	X		X	X			X		18	2535.18	7.33	0.00	2499.13	1.00
X	X	X	X	X	X			X	X		X	X				X	X	X	X	X		X	X			X		19	2536.95	9.11	0.00	2498.90	1.00
X	X	X	X	X	X	X		X	X		X	X				X	X	X	X	X		X	X			X		20	2538.85	11.01	0.00	2498.80	1.00

Supplementary Table 14. Model selection table for predictors of survival of lead mortality (S_{Pb}) for California condors (*Gymnogyps californianus*). Shown are model forms and AICc values for the top 20 known fate models to predict survival of lead hazards. Shaded models have higher ranked models nested within and thus contain parameters likely to be uninformative. Data consisted of near-daily records for all free-flying condors in the *Central* (CECA) and *Southern* (SOCA) flocks in California from *Bioyear* 1996 through 2022, or 1452 annual records for 226 condors and 61 deaths in *Central* and 1428 annual records for 226 condors and 32 deaths in *Southern*. Model results shown in Supplementary Table 6b are from the second -ranked model here, selected because it receives similar support to top-ranked model but provides more information on processes driving survival changes in the *Post19Ban* time interval. See Supplementary Table 1 for variable definitions. Other abbreviations: K is number of parameters.

First	Young		Coastal	Proffered			Presence		Post08Ban		Post19Ban		DeerHunt		PigHunt		Post08Ban: DeerHunt		Post19Ban: DeerHunt		Post08Ban: PigHunt		Post19Ban: PigHunt		K	AICc	delta AICc	AICc weight	Deviance	Cumul. weight
2Years	Adult	CECA	CECA	--	CECA	SOCA	CECA	SOCA	CECA	SOCA	CECA	SOCA	CECA	SOCA	CECA	SOCA	CECA	SOCA	CECA	SOCA	CECA	SOCA	CECA	SOCA						
X	X		X	X							X							X			X				8	1081.43	0.00	0.20	1065.42	0.20
X	X		X	X														X			X		X		8	1081.82	0.39	0.16	1065.82	0.36
X	X		X	X							X					X		X			X				9	1082.64	1.21	0.11	1064.63	0.47
X	X	X	X	X							X					X		X			X				10	1082.87	1.43	0.10	1062.85	0.57
X	X	X	X	X						X	X					X		X			X				11	1082.94	1.51	0.09	1060.93	0.66
X			X	X							X							X			X				7	1083.22	1.79	0.08	1069.21	0.74
X	X		X	X							X							X			X		X		9	1083.39	1.96	0.07	1065.38	0.82
X	X	X	X	X				X	X	X	X				X	X		X							13	1084.03	2.60	0.05	1058.01	0.87
X	X	X	X	X				X		X	X							X			X				11	1084.25	2.82	0.05	1062.23	0.92
X	X	X	X	X				X	X	X	X				X	X		X						X	14	1085.99	4.55	0.02	1057.96	0.94
X	X		X	X							X										X				7	1086.26	4.82	0.02	1072.25	0.96
X	X	X	X	X				X	X	X	X	X			X	X		X						X	15	1086.48	5.05	0.02	1056.45	0.97
X	X	X	X	X			X	X	X	X	X	X			X	X		X						X	16	1087.42	5.99	0.01	1055.39	0.98
X	X		X	X					X		X							X							8	1087.48	6.04	0.01	1071.47	0.99
X	X	X	X		X	X	X	X	X	X	X	X			X	X		X						X	17	1088.76	7.32	0.01	1054.72	1.00
X	X	X	X		X	X	X	X	X	X	X	X	X		X	X		X						X	18	1090.55	9.11	0.00	1054.51	1.00
X	X	X	X		X	X	X	X	X	X	X	X	X		X	X		X	X					X	19	1092.15	10.72	0.00	1054.11	1.00
X	X	X	X		X	X	X	X	X	X	X	X	X		X	X		X	X	X				X	20	1093.98	12.55	0.00	1053.93	1.00
X	X	X	X		X	X	X	X	X	X	X	X	X		X	X		X	X	X		X		X	21	1095.97	14.54	0.00	1053.91	1.00
X	X	X	X		X	X	X	X	X	X	X	X	X	X	X	X		X	X	X		X		X	22	1097.96	16.53	0.00	1053.90	1.00

Supplementary Table 15. Model for reproduction of California condors (*Gymnogyps californianus*) in California and Baja California, Mexico used in population model. Beta coefficients for predictors of a female successfully fledging a chick, *Succ*, for California condors in the *Central*, *Southern*, and *Baja* flocks using GLMMs. See Supplementary Table 1 for variable definitions.

Fixed effect	Coefficient Estimate	Std. Error	z value	Pr(>|z|)
Intercept	-9.383	3.189	-2.942	0.003
EarlyBreedingYrs	-2.071	0.768	-2.696	0.007
Age5	-2.088	1.095	-1.906	0.057
Ageto9	0.310	0.154	2.015	0.044
FreeFly	5.279	2.968	1.779	0.075
SuccessfulBreeder	-1.397	0.491	-2.845	0.004
FailedBreeder	0.524	0.356	1.472	0.141
**Random effect**				
Groups	**Variance**			
ID	0.604			
Number of obs:	780			
ID	129			

Supplementary Table 16. Survival model for California condors (*Gymnogyps californianus*) in California and Baja California, Mexico used in population model. Beta coefficients for predictors of $S_{Overall}$ for California condors in the *Central* (CECA), *Southern* (SOCA), and *Baja* (BACA) flocks using known fate models based on near-daily records for all free-flying condors for *Bioyear* 1996 through 2022, or 1452 annual records for 226 condors in CECA, 1428 annual records for 226 condors in SOCA, and 585 annual records for 83 condors in BACA. See Supplementary Table 1 for variable definitions.

Effect	Coefficient Estimate	Std. Error	Lower 95	Upper 95
Intercept	2.642	0.100	2.446	2.839
FirstYear	-0.685	0.244	-1.162	-0.207
Released<1yr	-0.609	0.167	-0.937	-0.281
BACA	0.654	0.225	0.213	1.095
Female	-0.337	0.127	-0.586	-0.088
BACA:Yr<6Yrs	-0.511	0.572	-1.632	0.609
CECA:Yr<6Yrs	1.844	1.030	-0.175	3.864
SOCA:Yr<6Yrs	-0.894	0.266	-1.415	-0.374

References

1. Bakker VJ, *et al.* Practical models to guide the transition of California condors from a conservation-reliant to a self-sustaining species. *Biol. Conserv.* **291**, 110447 (2024).
2. Kurle CM, *et al.* Terrestrial scavenging of marine mammals: cross-ecosystem contaminant transfer and potential risks to endangered California condors (*Gymnogyps californianus*). *Environ. Sci. & Technol.*, 9114-9123 (2016).
3. Bakker VJ, *et al.* Effects of lead exposure, flock behavior, and management actions on the survival of California condors (*Gymnogyps californianus*). *EcoHealth* **14**, 92-105 (2017).
4. Zens MS, Peart DR. Dealing with death data: individual hazards, mortality and bias. *Trends. Ecol. Evol.* **18**, 366-373 (2003).
5. Galipaud M, Gillingham MAF, Dechaume-Moncharmont F-X. A farewell to the sum of Akaike weights: The benefits of alternative metrics for variable importance estimations in model selection. *Methods Ecol. Evol.* **8**, 1668-1678 (2017).
6. Cade BS. Model averaging and muddled multimodel inferences. *Ecology (Wash D C)* **96**, 2370-2382 (2015).

Any use of trade, firm, or product names is for descriptive purposes only and does not imply endorsement by the U.S. Government.
