# Supplementary material for: Behavioral shifts mask the success of legislation and outreach for endangered species recovery
Source: Nat Commun. 2026 Mar 18;17:1819. doi: 10.1038/s41467-026-69617-4 (PMC13000190; doi:10.1038/s41467-026-69617-4)
Supplement: Supplementary file 2 — Description of Additional Supplementary Files [file 41467_2026_69617_MOESM2_ESM.pdf]

Description of Additional Supplementary Files

File name: Supplementary Data 1

Description: Input data and code. Includes:

1. InputDataCACO.xlsx. Excel file containing input data needed to run all analyses. Data are included in separate tabs:
    - VarDefs (variable definitions for all data in xlsx file),
    - PbDataCaMx (blood lead levels for Central, Southern and Baja),
    - PbFeatherDataCaMx (feather lead levels for Central, Southern and Baja),
    - PbDataCa (blood lead levels for Central and Southern, with covariates),
    - sAllCaMx (input data for overall survival, sOverall, analyses for Central, Southern and Baja),
    - PbCaMx (input data for lead survival, sPb, analyses for Central, Southern and Baja),
    - sOtherKnownCaMx (input data for survival of other known causes of death to correct sPb analyses for Central, Southern and Baja),
    - sMIWUndCaMx (input data for survival of unknown causes of death to correct sPb analyses for Central, Southern and Baja),
    - sAllCa (input data for overall survival, sOverall, analyses for Central and Southern, with covariates),
    - sPbCa (input data for lead survival, sPb, analyses for Central and Southern, with covariates)
    - reproAll (input data for reproductive rate analyses for Central, Southern, and Baja),
    - DeerHuntBiMo (bimonthly deer tag reports),
    - PigHuntBiMo (bimonthly pig tag reports,
    - PigCullMo (monthly pig cull reports),
    - Nonlead (nonlead outreach effort data)
2. PbAndSurvBasicTrends.Rmd. R notebook that calculates basic trends in blood lead exposure by flock and time period and feather lead by flock and basic trends in overall and lead survival by flock and year.
3. PbExposureLmmAnalyses.Rmd. R notebook that runs all linear mixed models analyzing drivers of lead exposure and also extrapolates effects of outreach on lead exposure to survival.
4. SurvReproPvaAnalyses.Rmd. R notebook that runs analyses of drivers of overall and lead survival. This notebook also estimates survival and reproduction to

parameterize age- and stagebased matrix models to generate population growth rates for the Southern, Central, and Baja flocks.

5. SetupPGJagsModels.Rmd. R notebook that sets up Poisson Gamma models to analyze meal contamination rates using the R script DoubleEquationplus.jags.condors.R.
6. DoubleEquationplus.jags.condors.R. R script that contains the jags code to run analyses of meal contamination rates for Southern and Central flocks using Poisson-Gamma model.
