# Supplementary material for: Behavioral shifts mask the success of legislation and outreach for endangered species recovery
Source: Nat Commun. 2026 Mar 18;17:1819. doi: 10.1038/s41467-026-69617-4 (PMC13000190; doi:10.1038/s41467-026-69617-4)
Supplement: Supplementary file 4 — Reporting Summary [file 41467_2026_69617_MOESM4_ESM.pdf]

nature portfolio

Corresponding author(s): Victoria J. Bakker, Myra E. Finkelstein

Last updated by author(s): 2025/12/02

Reporting Summary

Nature Portfolio wishes to improve the reproducibility of the work that we publish. This form provides structure for consistency and transparency in reporting. For further information on Nature Portfolio policies, see our Editorial Policies and the Editorial Policy Checklist.

Statistics

For all statistical analyses, confirm that the following items are present in the figure legend, table legend, main text, or Methods section.

n/a	Confirmed	
☐	☒	The exact sample size (*n*) for each experimental group/condition, given as a discrete number and unit of measurement
☐	☒	A statement on whether measurements were taken from distinct samples or whether the same sample was measured repeatedly
☒	☐	The statistical test(s) used AND whether they are one- or two-sided<br>*Only common tests should be described solely by name; describe more complex techniques in the Methods section.*
☐	☒	A description of all covariates tested
☐	☒	A description of any assumptions or corrections, such as tests of normality and adjustment for multiple comparisons
☐	☒	A full description of the statistical parameters including central tendency (e.g. means) or other basic estimates (e.g. regression coefficient) AND variation (e.g. standard deviation) or associated estimates of uncertainty (e.g. confidence intervals)
☒	☐	For null hypothesis testing, the test statistic (e.g. F, t, r) with confidence intervals, effect sizes, degrees of freedom and P value noted<br>*Give P values as exact values whenever suitable.*
☐	☒	For Bayesian analysis, information on the choice of priors and Markov chain Monte Carlo settings
☐	☒	For hierarchical and complex designs, identification of the appropriate level for tests and full reporting of outcomes
☒	☐	Estimates of effect sizes (e.g. Cohen's *d*, Pearson's *r*), indicating how they were calculated

Our web collection on statistics for biologists contains articles on many of the points above.

Software and code

Policy information about availability of computer code

Data collection	not applicable
Data analysis	All analyses were conducted using the R environment for statistical computing (version 4.4.1, https://www.r-project.org/), with the exception of spatial data summaries, which were conducted in ArcGIS Pro 3.3.1. Packages used are provided in the Methods or in the code. All code is included with the Supplementary Information.

For manuscripts utilizing custom algorithms or software that are central to the research but not yet described in published literature, software must be made available to editors and reviewers. We strongly encourage code deposition in a community repository (e.g. GitHub). See the Nature Portfolio guidelines for submitting code & software for further information.

Data

Policy information about availability of data

All manuscripts must include a data availability statement. This statement should provide the following information, where applicable:

- Accession codes, unique identifiers, or web links for publicly available datasets
- A description of any restrictions on data availability
- For clinical datasets or third party data, please ensure that the statement adheres to our policy

All data required to replicate analyses and produce figures presented in this study are provided in Supplementary Information as Supplementary Data 1. These include data on condor behavior and blood lead levels as well as data on public outreach effort and deer and pig tag returns. The spatial data used to identify high-

use foraging counties for the Southern California flock were collected by the U.S. Fish and Wildlife Service and are publicly available on ScienceBase [https://www.sciencebase.gov/catalog/item/546f5ec5e4b0b935bc7586e0available] while those for the Central California flock were collected by Ventana Wildlife Society and Pinnacles National Park and are under restricted access due to endangered species management policy. Qualified researchers may obtain these data by contacting the corresponding author and agreeing to data sharing restrictions. Data from the California Protected Areas Database are available at https://data.cnra.ca.gov/dataset/california-protected-areas-database. This is a GIS database consisting of lands protected for open space by over 1,000 public agencies or non-profit organizations and maintained and published by GreenInfo Network (www.greeninfo.org).

Research involving human participants, their data, or biological material

Policy information about studies with human participants or human data. See also policy information about sex, gender (identity/presentation), and sexual orientation and race, ethnicity and racism.

Reporting on sex and gender	not applicable
Reporting on race, ethnicity, or other socially relevant groupings	not applicable
Population characteristics	not applicable
Recruitment	not applicable
Ethics oversight	not applicable

Note that full information on the approval of the study protocol must also be provided in the manuscript.

Field-specific reporting

Please select the one below that is the best fit for your research. If you are not sure, read the appropriate sections before making your selection.

☐ Life sciences ☐ Behavioural & social sciences ☒ Ecological, evolutionary & environmental sciences

For a reference copy of the document with all sections, see nature.com/documents/nr-reporting-summary-flat.pdf

Ecological, evolutionary & environmental sciences study design

All studies must disclose on these points even when the disclosure is negative.

Study description	We used condor spatial, behavioral, health, reproductive, and mortality data for all free-flying California condors in California and Mexico as well as data on public outreach efforts and hunter tag returns and cull reports to identify factors predicting condor blood lead levels and survival, estimate population growth rates, and estimate meal contamination rates.
Research sample	We analyzed data for all free-flying condors in the Central and Southern flocks in California and all condors in the Baja flock in Mexico.
Sampling strategy	All free-flying condors and offspring that survived to fledging were known, representing a complete population census.
Data collection	Condor behavioral and biological data were collected as part of routine monitoring of this endangered species and provided for this study. Near daily data have been collected continuously for all condors across their range since the California condor release program was initiated in 1992.
Timing and spatial scale	We analyzed data for all free-flying condors in the Central and Southern flocks in California and all condors in the Baja flock in Mexico from Sep 1996 through Aug 2023.
Data exclusions	We excluded data from 1992 to 1996, when flock sizes were extremely small and condors were frequently moved in and out of captivity. We also excluded condors in the newly established flock in northern California, where releases started in 2022.
Reproducibility	Data and code to reproduce these analyses are provided as Supplemental Information.
Randomization	All free-flying condors in California were included in study.
Blinding	not applicable

Did the study involve field work? ☐ Yes ☒ No

Reporting for specific materials, systems and methods

We require information from authors about some types of materials, experimental systems and methods used in many studies. Here, indicate whether each material, system or method listed is relevant to your study. If you are not sure if a list item applies to your research, read the appropriate section before selecting a response.

Materials & experimental systems

n/a	Involved in the study
☒	☐ Antibodies
☒	☐ Eukaryotic cell lines
☒	☐ Palaeontology and archaeology
☐	☒ Animals and other organisms
☒	☐ Clinical data
☒	☐ Dual use research of concern
☒	☐ Plants

Methods

n/a	Involved in the study
☒	☐ ChIP-seq
☒	☐ Flow cytometry
☒	☐ MRI-based neuroimaging

Animals and other research organisms

Policy information about studies involving animals; ARRIVE guidelines recommended for reporting animal research, and Sex and Gender in Research

Laboratory animals

not applicable

Wild animals

All California condors (Gymnogyps californianus) in the Central and Southern flocks in California and in the Baja flock in Mexico were knowns and monitored. Attempts were made to capture all condors in baited field flight pens once or twice annually as part of ongoing monitoring and management of this endangered species. Condors were released alive from capture locations unless management or medical needs required relocation to another release site or captivity for medical care. No animals were killed.

Reporting on sex

Results apply to both sexes.

Field-collected samples

not applicable

Ethics oversight

California condor (Gymnogyps californianus) behavioral data and biological samples (blood, feathers) were collected and analyzed according to all relevant ethical regulations and permit authorizations. Specifically, personnel affiliated with the California Condor Recovery Program collected behavioral data and biological samples as part of routine monitoring of condors in California, USA and Baja California, Mexico under appropriate state and federal permits as follows: Hopper Mountain National Wildlife Refuge Complex Federal permit: ES108507 (previously 02360A HMNWRC), California state permit: 2081a-2014-047-00; Ventana Wildlife Society Federal permit: TE 026659, California state permit: 2081a-2014-054-00; Pinnacles National Park Federal permit: ES157291-3, California state permit: 2081a-2014-048-00, Cruz Institutional Animal Care and Use Committee (IACUC) through the National Park Service Biological Resources Division: CA_PINN_Welch_Condor_2021.A3; Parque Nacional Sierra de San Pedro Mártir: Federal authorization from the Secretaría de Medio Ambiente y Recursos Naturales (SEMARNAT) of Mexico. California condor biological samples were received and analyzed with the University of California Santa Cruz IACUC approval under the following protocols: Smitd0712, Finkm1102, FINKM1307, FINKM1607, Finkm1907, Finkm2207dn.

Note that full information on the approval of the study protocol must also be provided in the manuscript.

Plants

Seed stocks

not applicable

Novel plant genotypes

not applicable

Authentication

not applicable
